# Bayesian Action–Perception Computational Model: Interaction of Production and Recognition of Cursive Letters

**DOI:** 10.1371/journal.pone.0020387

**Published:** 2011-06-01

**Authors:** Estelle Gilet, Julien Diard, Pierre Bessière

**Affiliations:** 1 Estelle Gilet Laboratoire d'Informatique de Grenoble, INRIA Rhône-Alpes, CNRS, Montbonnot, France; 2 Julien Diard Laboratoire de Psychologie et NeuroCognition, CNRS, Université Pierre-Mendès-France, Grenoble, France; 3 Pierre Bessière Laboratoire d'Informatique de Grenoble, INRIA Rhône-Alpes, CNRS, Montbonnot, France; 4 Laboratoire de Physiologie de la Perception et de l'Action, Collège de France, CNRS, Paris, France; Indiana University, United States of America

## Abstract

In this paper, we study the collaboration of perception and action representations involved in cursive letter recognition and production. We propose a mathematical formulation for the whole perception–action loop, based on probabilistic modeling and Bayesian inference, which we call the Bayesian Action–Perception (BAP) model. Being a model of both perception and action processes, the purpose of this model is to study the interaction of these processes. More precisely, the model includes a feedback loop from motor production, which implements an internal simulation of movement. Motor knowledge can therefore be involved during perception tasks. In this paper, we formally define the BAP model and show how it solves the following six varied cognitive tasks using Bayesian inference: i) letter recognition (purely sensory), ii) writer recognition, iii) letter production (with different effectors), iv) copying of trajectories, v) copying of letters, and vi) letter recognition (with internal simulation of movements). We present computer simulations of each of these cognitive tasks, and discuss experimental predictions and theoretical developments.

## Introduction

This paper concerns the study of the cognitive processes involved in perception and action, and, more precisely, in the tasks of reading and writing. Although these are ubiquitous in everyday life, there is no consensus as to the principles and processes underlying them.

More precisely, we would argue that these dual tasks of reading and writing have, surprisingly, seldom been studied jointly.

Although much recent evidence outlines their interaction, using both behavioral studies [1]–[4] and neuroimaging studies [5], [6], most previous studies have focused on either models of movement production or systems of handwriting recognition.

Studies of human motor control, for instance, have commonly focused on open-loop control; that is, they have considered tasks where perceptive feedback was suppressed, or highly controlled [7]–[9]. However, even though it is intuitive that drawing a single letter can be performed in an open-loop manner, the consequent readability of the produced trajectory has never been taken into account.

Purely sensory models usually describe letters in some image-based space, which might, in turn, be difficult to use as a basis for movement planning and production [10], [11]. This is a common and justified approach in the case of the design of industrial systems dealing, for instance, with optical character recognition (OCR). However, it severely limits the plausibility of these methods as viable models of the human cognitive systems involved in reading and writing.

**Figure 1:**
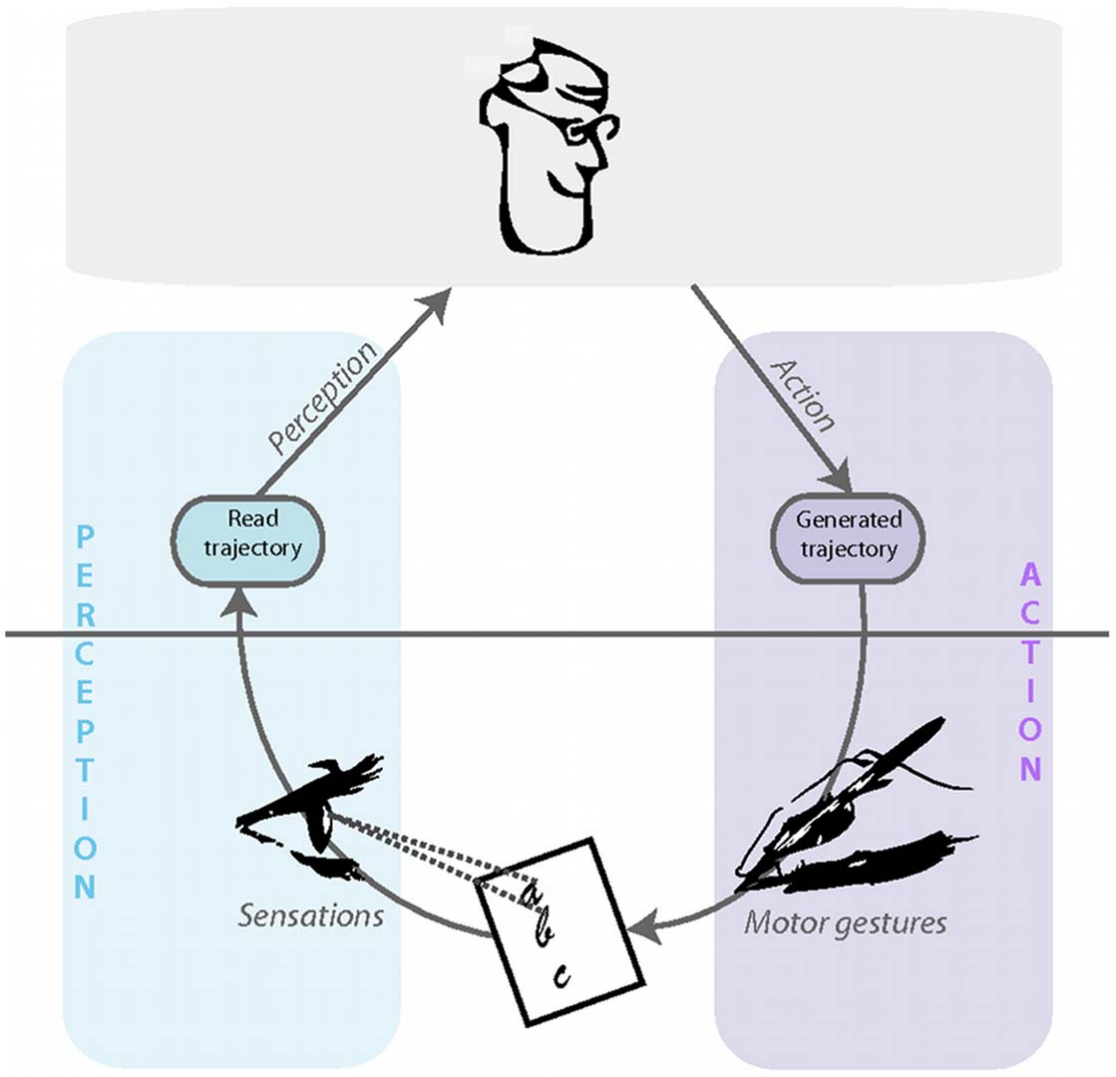
General structure of the BAP model. Handwriting and reading are studied as a perception–action loop.

We argue that most of the above approaches are hemiplegic in nature; we thus propose studying reading and writing as parts of a complete perception and action loop (see Fig. 1).

In this context, we develop and present a mathematical model of this perception–action loop. It is based on a probabilistic framework. More precisely, we apply the Bayesian Programming methodology [12], [13], in which complex models are built using probability distributions and their combinations, and in which Bayesian inference is systematically used to solve and simulate cognitive tasks. We call our model the Bayesian Action–Perception (BAP) model [14].

In this model of perception and action, it is possible to study the recognition of letters and their production, and the interaction between perception and action. The first side of this interaction concerns the influence that the prediction of future perceptions has on the current choice of action: this is the classical problem of modeling closed-loop control, which has already received a lot of attention. We thus focus instead on the second side of this interaction, that is, we are interested in the influence of motor knowledge on perception. To capture this influence, the BAP model includes an internal motor simulation loop, which may be recruited in perception tasks.

We will show that the BAP model solves a wide variety of cognitive tasks related to reading and writing. We simulate six cognitive tasks: i) letter recognition (purely sensory), ii) writer recognition, iii) letter production (with different effectors), iv) copying of trajectories, v) copying of letters, and vi) letter recognition (with internal simulation of movements).

As our goal is the study of the interaction of action and perception, we restrict ourselves to the case of isolated letters to limit lexical, semantic and other top-down effects related to the global perception of words. Furthermore, we treat the case of online recognition, where the presented trajectories contain both spatial and sequence information. In other words, we consider perception tasks where the letter is perceived as it is being traced.

The remainder of this paper is structured as follows. First, we detail the founding hypotheses of our approach and define the overall structure of the BAP model. We then give the corresponding mathematical formulation, using the Bayesian Programming methodology. Once the model is defined, we show how it is used to solve our six cognitive tasks automatically using Bayesian inference.

## Methods

### BAP model: assumptions and model architecture

The first and main hypothesis we make is that an internal representation 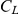 is associated with each letter 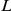 and each writer 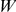. Therefore, we encode, using terms in the form 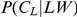, probability distributions for the representation of letters, given the letter and writer under consideration. Moreover, we assume that these representations act as pivots between perception (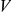, for vision) and action (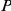, for production). In other words, perception and action are assumed to be independent, conditionally on the knowledge of the representation of a letter 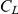. This can be seen as the probabilistic translation of the common-coding approach to perception and action [15]. This yields the following joint probability distribution over this set of variables:



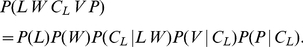
(1)This describes the overall architecture, and the heart of our model. To detail its definition and structure further, we make four main hypotheses.

- There are two connected internal representations of letters.
- Letters are encoded in a Cartesian workspace.
- Letters are encoded by sequences of via-points (set at cusps and points of the trajectory where the tangent is either vertical or horizontal).
- The motor system influences perception via internal simulation of movements.

### Two connected internal representations of letters

Should an internal representation be common to perception and action? This has been widely studied in the cognitive science literature, in particular in the speech perception community, where purely auditory theories of perception have been long debated. For instance, the motor theory of speech perception [16], [17] claims that perceiving speech amounts to identifying vocal tract gestures rather than sound patterns. In this case, it is assumed that there is a single internal representation, shared by perception and action, that is purely motor in nature. On the other hand, the Perception-for-Action-Control Theory (PACT) [18] proposes that, instead of a single representation, which has to be either perceptual or motor, there are two linked internal representations. These perceptual and motor representations then constrain each other as they are acquired together through experience.

**Figure 2:**
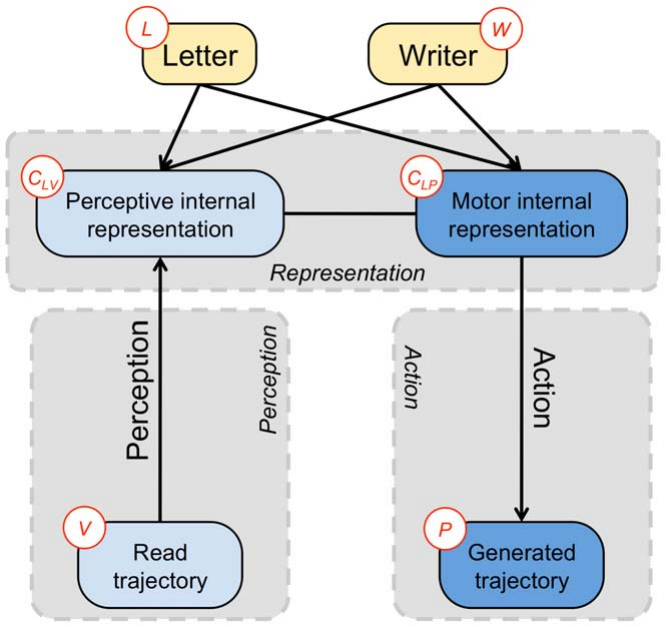
The representation of letters is the pivot between perception and action. The perception model links the internal representation with the read trajectory. The action model links the internal representation with the written trajectory. Variables: 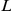 letter, 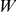 writer, 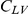 perceptive internal representation, 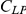 motor internal representation, 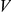 read trajectory, 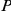 generated trajectory. The corresponding joint probability distribution is defined Eq. (2).

In the BAP model, we assume, as in PACT, that there are two distinct representations of letters: 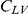 is the (visual) perceptual representation, and 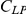 is the (production) motor representation (see Fig. 2). During computations, this allows them to be activated with different values simultaneously. However, they are encoded in the same space and in the same manner, as in the common-coding approach.

We thus refine the decomposition of the joint distribution as follows:



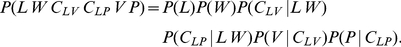
(2)


### Letter encoding in the Cartesian workspace

If you were asked to write down your name, you would probably consider it a mundane task. You could surely perform it easily in a variety of circumstances, like thinking about something else, or looking elsewhere. But what about writing your name with your foot, in the sand or snow, for instance? It turns out that this, too, is rather easy. The performed trace would be somewhat distorted from your handwriting, but, even without any training in “footwriting”, your name would be readable. Moreover, the characteristics of your handwriting would also be found and be recognizable in the trajectory you perform with your foot.

This effect is known as *motor equivalence* [19]–[21]. It has been used as evidence that internal representations of movements might be independent of the effector usually used to perform them.

**Figure 3:**
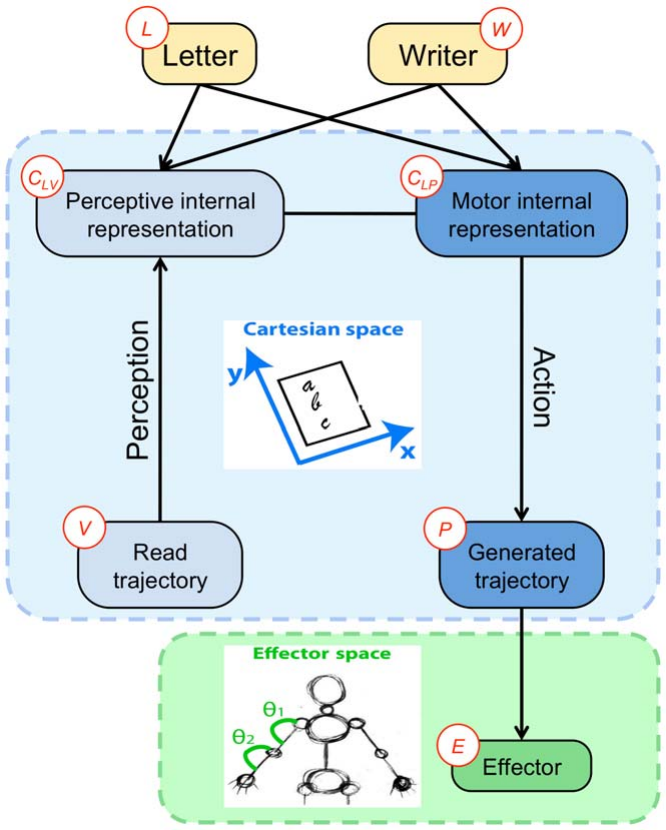
General structure of the BAP model, including the effector model. The input trajectory, perceptive and internal motor representation and generated trajectory are defined in Cartesian space (circled in blue) and the effector model is defined in joint space (circled in green). Variables: 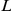 letter, 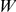 writer, 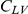 perceptive internal representation, 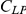 motor internal representation, 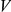 read trajectory, 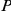 generated trajectory, 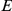 effector. The corresponding joint probability distribution is defined Eq. (3).



In the BAP model, we thus assume that the internal representation of letters is described in the task space: i.e., the Cartesian space or workspace. Fig. 3 specifies the space of each submodel. We add the effector model 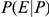 into the model:

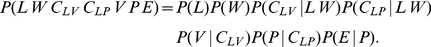
(3)


### Letter encoding by sequences of via-points

There is a strong dichotomy in the literature between representations of letters that are tailored for handwriting recognition and those tailored for handwriting production. These two domains have given rise to types of letter representations that are very different in nature.

Firstly, consider handwriting recognition. Most approaches to character recognition have focused on probabilistic models and neuromimetic methods [10], [11], which consider various kinds of features of letter trajectories. These can be local features along the trajectory (peak, loop, pen-up) or global features, taking into account characteristics of the whole letter shape (height/width ratio, center of mass, etc.). Such varied features can be successfully combined using Hidden Markov Models (HMM) [22]–[24] or neuromimetic methods, based on artificial neural networks [25].

The most successful methods, which usually are combinations of these techniques, achieve low misclassification rates (between 5 and 10%).

Secondly, let us turn to handwriting production. Many models have already been proposed to tackle the problem of handwriting generation. For instance, in the classical mass–spring model, handwriting arises from orthogonal oscillations in the plane of the writing surface [7].

Another, large class of models considers trajectories to be summarized by a small set of points in the 2D plane. These can be outside of the trajectory, as in classical spline interpolation, and are then usually called control points, or are restricted to being along the trajectory, and are then usually called *via-points*.

For instance, trajectories can be assumed to be the concatenation of elementary strokes [8]. Handwritten trajectories are then planned using simple segments, with a repertoire of four segments being sufficient to produce any cursive character. Each segment is planned with a minimum-jerk extended model, and these are connected with via-points. In contrast to the stroke-by-stroke trajectory generation used in the FIRM model [26], [27], via-points are set using an iterative algorithm that minimizes the spatial discrepancy between a proposed trajectory and a goal trajectory.

Finally, turning back to the representation of letters in BAP, we would argue that none of the previous representations is suitable for our purpose. Indeed, our aim is to tackle, using the same representation, both letter recognition and letter production. We therefore need to choose a letter representation that can be presumed to be relevant for both processes and that thus has clear a priori semantics.

Consider, for instance, the global features used to help character recognition in HMM methods: these would be difficult to use as guides during trajectory generation. Similarly, in the mass–spring model, recovering the parameters of a mass–spring system that generates a trajectory appears to be nontrivial, even though any trajectory can be generated given mass and spring parameters. Finally, although via-points were shown to be sufficient for recognition purposes in the FIRM model, their semantics had to be explored experimentally. Even though it appears that via-points were mostly placed at vertical velocity zero-crossings, and also approximately between vertical velocity zero-crossings, this is by no means systematic (see, for instance, the bottoms of the *b*, *d* and *g* of Fig. 7 of [27]).

**Figure 4:**
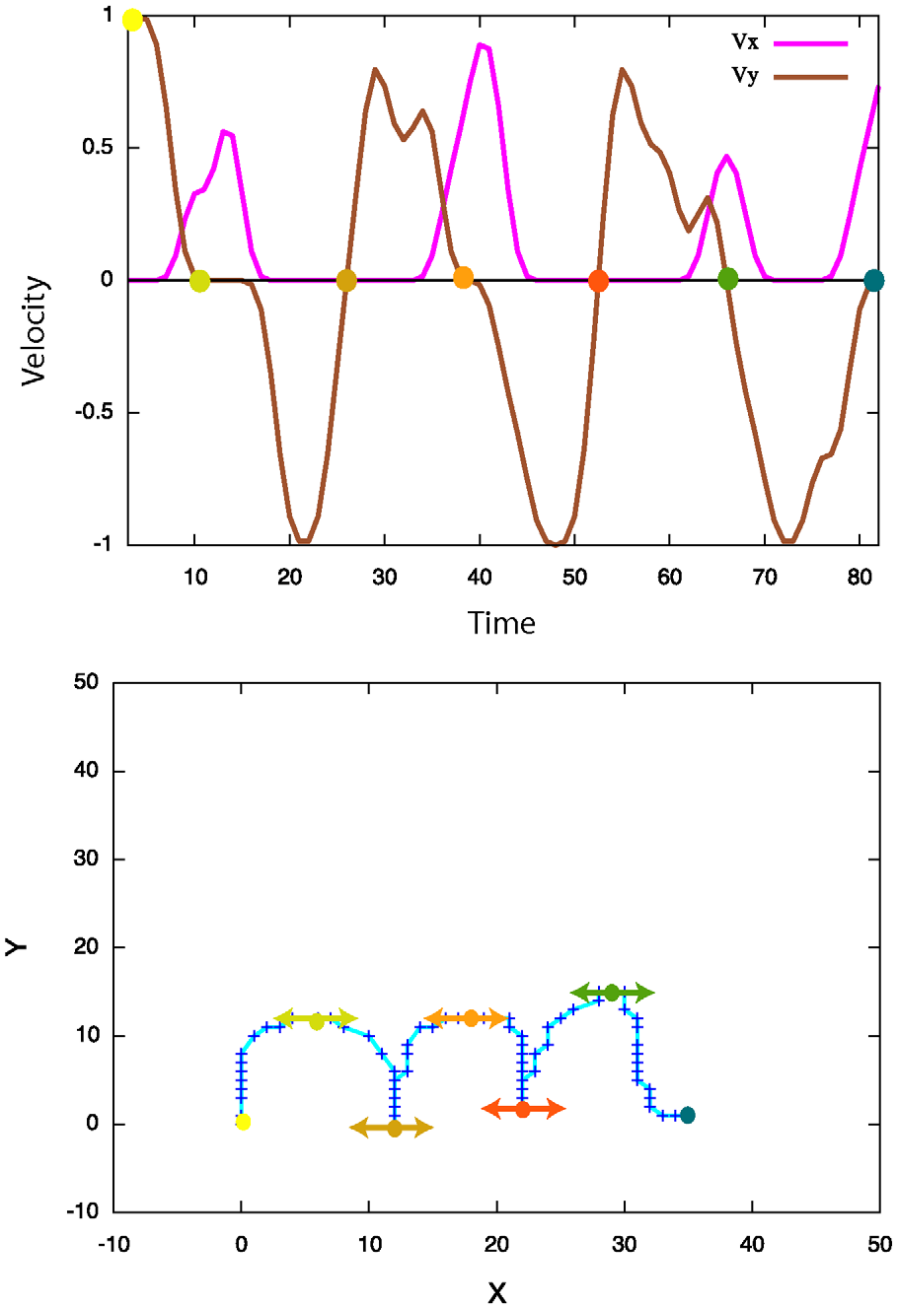
Example of positions of via-points on a letter. Top: Velocity (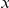 and 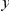) profiles corresponding to the letter *m* shown below. Via-points are placed where one of the velocities is zero (dots on the 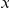-axis, top), which corresponds to horizontal or vertical tangents (colored arrows, bottom).

As with this last model, we firstly assume that letters are represented by a sequence of via-points, and we place them where either the 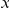 derivative (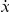) or the 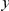 derivative (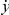), or both, is zero. In other words, via-points lie where tangents are horizontal or vertical, or at cusps. We also place via-points at the start and end positions of the trajectories, where tangents can follow arbitrary directions. Fig. 4 presents an example of via-points on a trajectory and the corresponding velocity profile.

On the one hand, via-points can easily be used as constraints for trajectory generation using some optimality criterion (see Section “Action model”) because they are placed at the zeroes of the velocities, and so they make sense from a control point of view. On the other hand, they also correspond to points in the trajectory having either vertical or horizontal tangents, so they are geometrically salient and make sense perceptively. Finally, such tangents, or at least the horizontal ones, are widely used by schoolteachers as constraints to be followed by children when they learn to produce letters correctly.

Of course, this makes our representation axis dependent: a letter always observed at an angle of 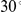 would have a different representation than if it was not slanted. However, there is evidence from mental rotation studies that suggests that letters are represented by humans in a canonical, upward orientation [28]. In our model, all data exemplars are assumed to be in such a canonical orientation before treatment.

We denote the set of via-points for a given trajectory as 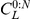. We set the maximum number of via-points to 16, which is quite sufficient for all trajectories considered in the remainder of this paper. Let 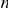 be an index in the sequence of via-points; each via-point is four dimensional:



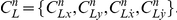
As the term 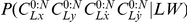 has a high dimensionality (64 dimensions for 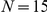), we use conditional independence hypotheses to decompose it into a product of smaller distributions. The joint distribution over this set of variables is defined as:



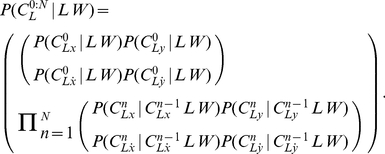
(4)


The product of terms indicates that positions and velocities are considered independent of each other if the letter and writer are known. Moreover, we assume that the positions and velocities of a via-point (index 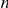) depend only on the positions and velocities of the previous via-point (index 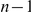); *i.e.*, it is a first-order Markov hypothesis.

The representation of letter model is complemented by two prior probability distributions over letters, 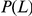 and writers, 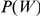: both are defined using uniform probability distributions, so that the model encodes no prior preference for any letter or writer.

This probabilistic model thus encodes, for each via-point of a letter, four probability distributions: two describe the positions of the via-point, and two describe the velocities of passages at that via-point. The mathematical forms for these distributions are set according to a learning process, that computes parameter values given a database of categorized trajectories: details are provided in the “Experimental data and parameter fitting” section.

### Influence of the motor system on perception via internal simulation of movements

Experimental observations suggest that the perceptions of performed actions are not only based on sensory cues, but also on internal simulations of actions [29], [30] when they are part of the action repertoire of the perceiving subject [31].

In the study of handwriting, a growing body of literature discusses the possible involvement of the motor system during the perception of letters, from behavioral studies [1]–[4] to neuroimaging investigations [5], [6].

For instance, the activation of motor areas of the brain during writing and reading tasks has been explored [5]. The main observation is that a part of the motor cortex is significantly activated during both tasks. This is surprising for the reading task: although the subjects stayed motionless, a motor area was activated. Another class of stimuli was presented: pseudoletters, which are as visually complex as letters, but for which the subjects had no previous experience writing. When such pseudoletters were visually presented, the same motor area was not activated.

A widely discussed interpretation of the above (and similar) observations is that perceiving a letter would entail a motor simulation of movements associated with the writing of that letter. This would, it is assumed, improve the perceptual recognition because of some, yet unexplained, mechanism.

**Figure 5:**
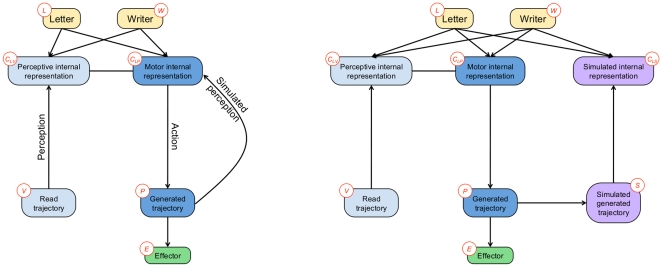
General structure of the BAP model, including a feedback loop. The feedback loop is added from planned trajectories to internal representations of letters, implementing an internal simulation of movements and simulated perception. Variables: 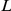 letter, 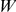 writer, 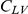 perceptive internal representation, 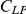 motor internal representation, 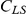 simulated internal representation, 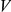 read trajectory, 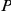 generated trajectory, 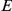 effector, 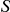 simulated generated trajectory. The corresponding joint probability distribution is defined Eq. (5).

In the BAP model, we propose a mathematical formulation of such a mechanism. The BAP model includes a formal model of motor simulation during perception: we add a feedback dependency along the production part of the model, from the planned trajectory back to the motor internal representation of letters (see Fig. 5, left). This dependency is a path back from simulated written letters to the internal representation: it is a simulated perception.

However, the feedback loop for internal simulation cannot be translated into the Bayesian framework directly. Probabilistic dependency structures cannot contain directed loops, otherwise they do not correspond to valid applications of Bayes' rule. The classical solution consists in duplicating nodes, *e.g.*, when temporal filters are modeled using Dynamic Bayesian Networks [32], [33]. In that case, the semantic of node duplication is temporal: copies of variables correspond to the same quantity, but at different points in time.

In our case, the desired semantic is to have two nodes for concurrently maintaining hypotheses about letters: one from perception and one from internal simulation of movements. Duplicating the whole production branch achieves this. This branch, with dependencies from planned trajectories back to internal representation of letters, corresponds to simulated perception. Therefore, it is defined exactly like the perception model. It encodes the same knowledge, except that, in this case, perceptual inputs are not external stimuli but are internally generated by the motor system: the internal representation 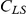 is extracted from the simulated written letter (probabilistic variable 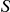). The resulting dependency structure (see Fig. 5, right) is:



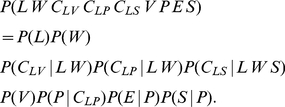
(5)


### BAP model: mathematical definition

So far, we have presented the four main hypotheses that define the overall architecture of the BAP model (Fig. 5). We will now detail each of these submodels in turn (perception, action, internal simulation). However, before that, we need to address a technical point: “Bayesian switches”.

### Bayesian switches

We introduce, in each of the three branches of perception, production and simulated perception, and between the two internal representations of letters, probabilistic switches in the form of 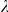 variables. These explicitly control the part of the model that is activated. When set (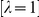), the submodel connected to 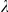 is activated. When treated as an unknown variable, the submodel is deactivated. Introducing 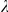 variables yields duplications of nodes on either side of them, where necessary. For instance, 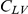 is duplicated into 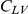 and 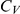 (see Appendix S1 for the complete mathematical definition of Bayesian switches).

**Figure 6:**
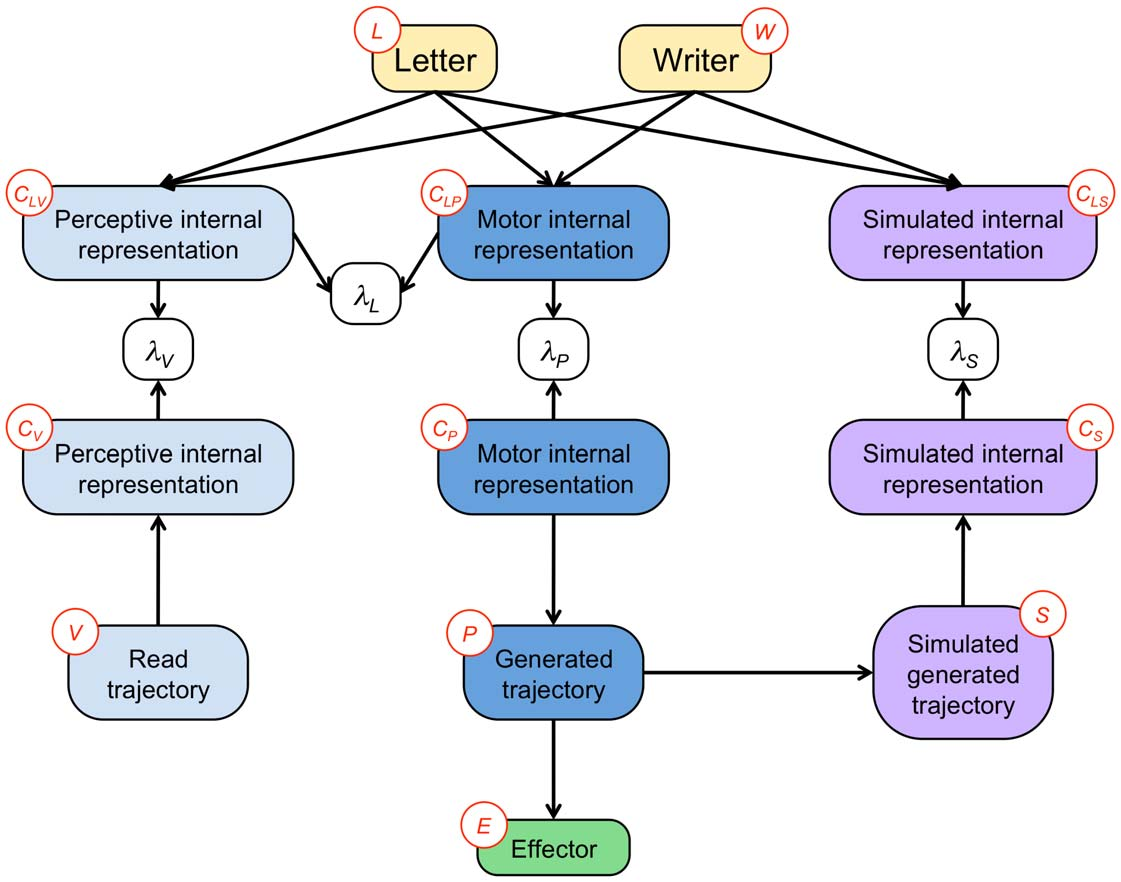
Global structure of the BAP model, including probabilistic switches. The probabilistic switches are represented by the 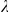 nodes. The model is composed of four main submodels: perception (left branch, in blue), action (middle, dark blue), simulated perception (right, purple) and letter representation (top, yellow), along with the effector model (bottom). Note that although the structure appears more complex than in Fig. 5, the difference is due to technical issues only, and the “semantic” structure is the same. Variables: 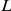 letter, 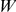 writer, 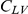 and 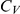 perceptive internal representations, 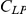 and 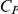 motor internal representations, 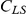 and 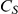 simulated internal representations, 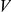 read trajectory, 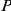 generated trajectory, 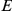 effector, 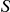 simulated generated trajectory, 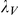, 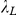, 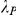 and 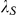 probabilistic switches. The corresponding joint probability distribution is defined Eq. (6).

The final dependency structure, with the 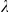 variables, is graphically displayed in Fig. 6, and it corresponds to the following decomposition of the joint probability distribution:



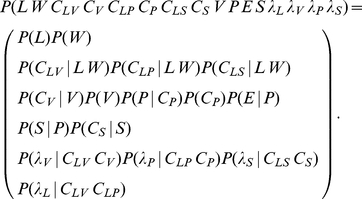
(6)


Recall that each term of the form 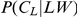 actually stands for a product of terms (see Eq. (4)); these are not shown here to improve readability.

At this point, some of the terms of Eq. (6) are already mathematically defined: 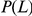, 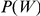, 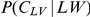, 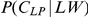, and 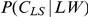 form the letter representation model, while 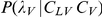, 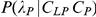, 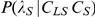 and 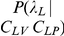 are the Bayesian switches. In the remainder of this section, we complete the mathematical definition of the model by providing the perception model 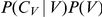, the action model 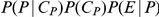 and the internal simulation model 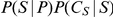.

### Perception model

The perception model concerns the collection and treatment of the sensory information. In our case, the stimuli are trajectories that are presented visually. However, because retinal projection and biologically plausible visual treatments are beyond the scope of our studies, we restrict our vision model to the simple task of extracting a sequence of via-points from the trajectory.

In Eq. (6), variable 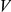 represents the visual input, which is encoded as a sequence of positions in the plane: 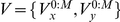 (for instance, obtained from a digital pen tablet), with 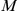 being the maximum number of points within the perceived trajectory. The term 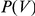 is a prior distribution, set as a uniform probability distribution, so as not to favor any visual input. The term 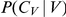 describes how the via-points are extracted from a trajectory. This follows from our via-point definition: when either or both derivatives of 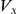 or 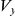 are zero, then a new via-point is found and the position and velocity profiles are encoded. We define the probabilistic term using Dirac probability distributions (delta functions), centered on the value given by our deterministic via-point extraction algorithm.

### Action model

The action model is concerned with the generation of movefments from the internal representations of letters. It is well known that visual feedback during movement execution plays a role [34], [35]. However, in the case of the generation of single cursive letters, which is a short-duration movement, we assume that such visual feedback can be safely neglected [36], and we consider movement generation as an open loop.

A widespread theory of movement production is optimal control theory [37], which assumes that, out of all possible movements to solve a task, the chosen one is optimal, in the sense that it minimizes some cost function. This cost can be defined either in the workspace (Cartesian) or in the articulatory joint space; depending on the chosen cost function, a variety of methods are obtained.

For instance, the square of the jerk (derivative of the acceleration) of the endpoint can be used, and the resulting trajectories are in good agreement with experimental human data [38]. In a similar way, criterion functions can be defined in articulatory space, such as the torque change generated by the actuator and the variance of the final arm position [39], [40]. Finally, the cost function is not necessarily a function of the geometry of the shape, but of the dynamics of its realization: a classical robotic control scheme (called bang-bang control) minimizes the time to travel from the initial configuration to the final one [41].

**Figure 7:**
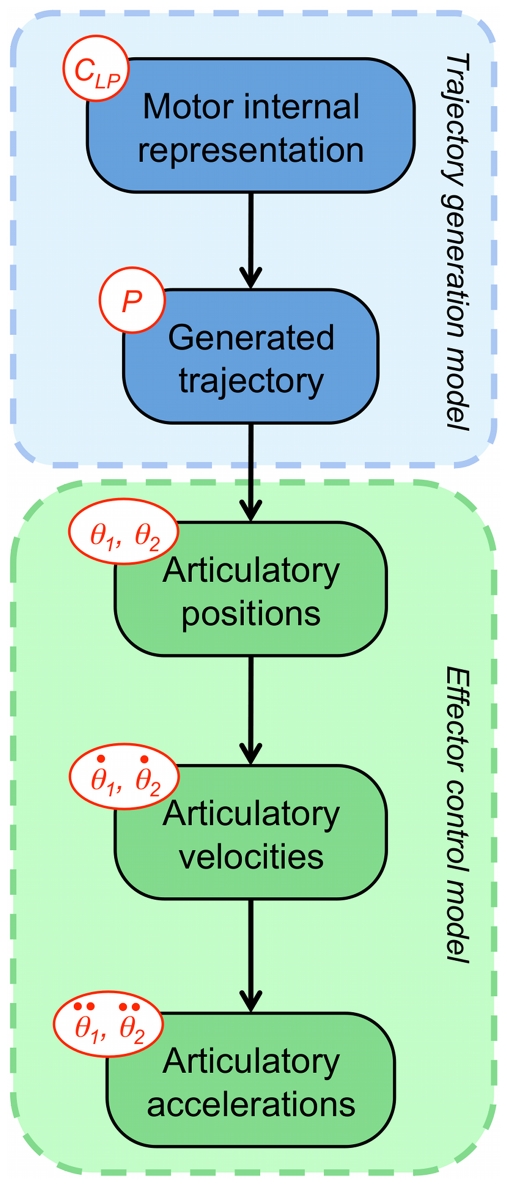
The action model is composed of two submodels: the trajectory generation and the effector model. The effector model is itself composed of three submodels: the inverse kinematics model and the velocity and acceleration models.

In the BAP model, writing movements are constrained in the 2D plane, so we assume that the cost of the control strategy is defined in the workspace [42]. Therefore, trajectory formation is independent of the effector used to perform the movement. This allows the action model to be decomposed into two submodels: the trajectory generation (or planning) model and the effector model (see Fig. 7).

Firstly, consider trajectory generation. Recall that via-points are defined as constraints of positions and velocities. The first free quantity that we can aim to minimize is the next derivative, that is to say, the acceleration. We therefore choose a minimum-acceleration model to generate trajectories. The cost function is:



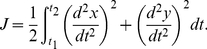
(7)


We define the limit constraints:



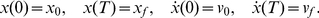
(8)


Using these constraints, we determine the following polynomial:



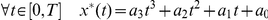


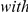


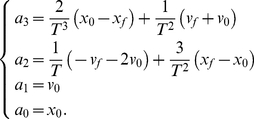
(9)


Returning to the probabilistic notation, we now define the action model. The first term is 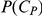, a uniform prior distribution over the position of via-points. The second term 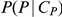 is concerned with general trajectory formation and is defined by Dirac probability distributions, centered on the solution provided by the above polynomial:



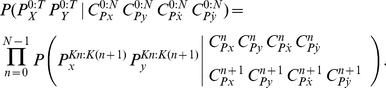
(10)The term inside the product describes the computation of 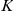 intermediary points between an initial position (current via-point 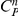) and a given destination position (the next via-point 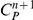). These points are spread evenly along the trajectory generated by the polynomial solution. In other words, Eq. (10) is a probabilistic encapsulation of the deterministic solution to trajectory generation of Eq. (9): this allows the BAP model to be uniformly defined in the probabilistic formalism. A resulting practical advantage is that, as the probabilistic inference engine is able to compute such deterministic portions of the model, there is no need to mix probabilistic inference with deterministic programming.

**Figure 8:**
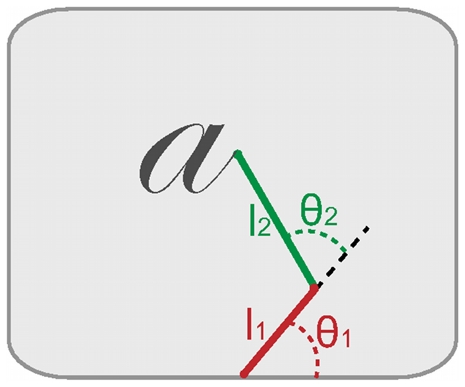
Schema of the two-joint manipulator used in our simulations. 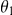
 represents the shoulder angle, and 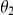 represents the elbow angle. The segment lengths are 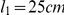 and 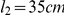, as in [39].



Secondly, we turn to the model of effector control. In our simulations, the human arm is represented by a two-joint manipulator (Fig. 8): 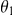 represents the shoulder angle, and 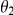 represents the elbow angle. The variable 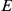 is the conjunction of joint positions, velocities and accelerations from time 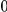 to 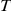: 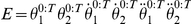. The endpoint position is described by its Cartesian coordinates 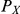 and 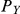. Therefore, the effector model is 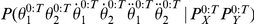. It is defined as a product of three terms:

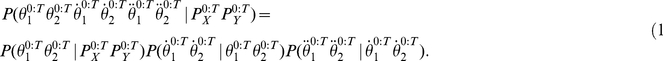
(11)


The first term, 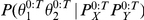, is based on the inverse kinematics transform, which translates the endpoint Cartesian coordinates to articulatory angles. The classical inverse kinematics solution for the two-joint manipulator gives 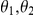 as functions of the endpoint position 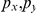 as follows [41]:



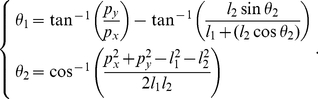
(12)The probability distribution over joint angles, 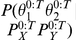, is a Dirac probability distribution centered on these values, at each point in time.

The second and third terms 
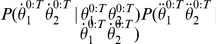
 concern the computation of successive derivatives using a finite difference method. More precisely, they allow probability distributions over velocities and accelerations to be computed, given the positions and velocities at time 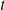 and 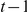.

### Internal simulation model

The variables of the motor simulation loop are 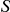, the simulated trajectory, 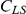, the simulated representation of letters, and its duplicate (for the probabilistic switch), 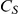.

The term 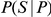 expresses the relationship between the simulated trajectory (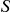), to be analyzed using simulated perception, and the generated trajectory (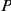). We define this term as an identity model: when simulated perception is activated, it takes a copy of the planned trajectory as an input. The term 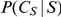 in the simulated perception is defined identically to the model of perception 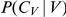: via-points are extracted from the planned trajectory using the same algorithm. Finally, the term 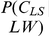 expresses the relationship between the simulated via-points, the writer and the letter.

### BAP model: simulation of cognitive tasks

The BAP model is now almost fully defined: the last step is to give its free parameters values. The only free parameters are in the internal representation of letters. This learning process in described in the “Results” section.

### Computation of probabilistic questions

Assuming the parameters are set, we here define and illustrate on an example the way the model is used in order to simulate cognitive tasks it can solve. This is done using Bayesian inference, in a systematic, automatized manner. Indeed, the BAP model defines a joint probability distribution over its variables, from which any probabilistic term of interest can be computed. This is demonstrated by the following theorem [13], [43].

Given a joint probability distribution over 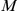 variables 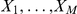, and given any partition of these variables into three subsets 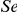, 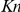, 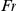 (for the searched, known and free variables, respectively), 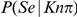 is computed from 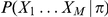 by:



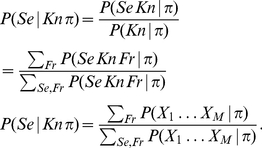
(13)The joint probability distribution is itself defined as a product of terms, so that any inference amounts to a number of sum and product operations on probability terms. Of course, this brute force inference mechanism sometimes yields impractical computation time ans space requirements, as Bayesian inference in the general case is NP-hard [44].

All of the inferences described in the remainder of the text have been carried out using a general purpose probabilistic engine, ProBT© from ProBayes. (The ProBT inference engine is available, free of charge, for academic purposes. Please refer to http://www.bayesian-programming.org/.) This inference engine uses two main phases to reduce computation time. The first is a symbolic simplification phase: it reorders the imbricated sums and products, and applies simplifications whenever possible. The second is a numerical computation phase, where most of the classical techniques are available, along with some custom methods for representation and maximization of probability distributions [45], [46].

Finally, since parts of the BAP model are based on Dirac probability distributions defined by deterministic functions, they can sometimes be extracted from the Bayesian inference equations. Our simulation algorithms are therefore defined in a fully probabilistic framework, and then implemented with combinations of deterministic and probabilistic programming.

Each probabilistic term 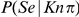 computed in this manner is called a *question*, and is associated with a cognitive task that is to be simulated and solved by the model. In the experimental section, we will present a series of cognitive tasks and describe how the model solves them. We now detail an example, based only on the letter representation part of BAP, in order to illustrate the general inference mechanism.

### Example: recognizing letters from via-point sequences

This example is preliminary to the case of letter recognition. In this simplified version, instead of a complete trajectory, we assume the BAP model is directly provided with the corresponding sequence of 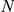 via-points 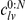, along with the identity 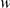 of the writer who generated the trajectory.

Given these via-points, we want the BAP model to compute the most likely letter they correspond to. In probabilistic terms, this is translated into the following question:



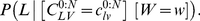
(14)This computes the probability distribution over letters, given the available input information. We now detail the inference and simplifications made in order to compute this question.

We first apply the general inference described by Eq. (13). In this case, the searched variable 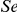 is 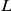, the known variables 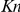 are 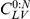 and 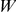, and the free variables 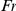 are all the other variables that appear in the BAP model, that is, 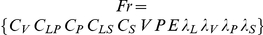 (see the decomposition, Eq. (6)). This yields:



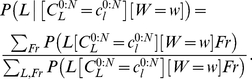
(15)The denominator is a constant, that does not depend on the value of 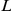. Therefore, instead of explicitly computing the large summation over 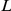 and 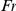, we can compute it afterwards as a normalization constant of the probability distribution over 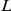. In other words, we first compute the result up to a proportionality constant 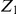, with:
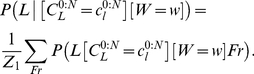
(16)Under the summation, we replace the joint probability distribution by the product of terms that define it (see Eq. (6)). This allows many symbolic simplifications, and yields:
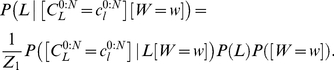
(17)Both terms 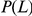 and 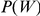 are defined by uniform probability distributions, so that their values are constants, independently of the value of 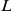. They can be included into the normalization constant which become 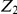:
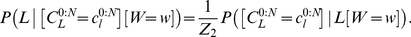
(18)Finally, recall that Eq. (4) specifies that 
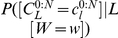
, the representation of letter model, is defined as a product of terms. This yields:



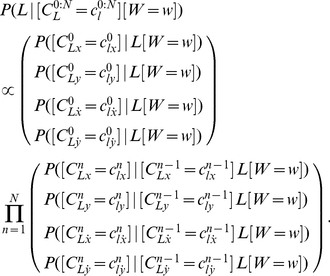
(19)


For each considered letter in 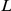, its probability is thus proportional to the product of 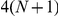 probability values (recall that 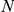 is the number of via-points). Once probabilities for all letters in 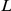 are computed, normalization yields the probability distribution over letters, given the input via-points and letter identity, which is the desired result.

## Results

### Experimental data and parameter fitting

#### Data collection

In order to set the parameters of the internal representation of letters, we have designed a data collection procedure and learning phase. Using a Wacom Intuos 3 pen tablet, we asked 4 adults to write 40 example trajectories of each of 22 letters, providing a complete database of 3,520 trajectories. We only considered letters without a pen-up movement for ease of data collection. The letters removed were *i*, *j*, *t* and *x*, as in [27].

### Parameter identification

The BAP model is now structurally completely defined. The only remaining piece to specify is the mathematical forms for the terms of the letter representation model (see Eq. (4). Except for the case of initial via-points, terms have the form 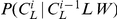, and encode information about the position and velocity of a via-point, given the previous via-point and letter and writer identity. Each of these probability distributions is defined as a Laplace succession law. For instance, for the 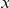 position 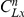:



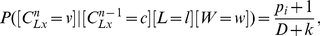
(20)


with 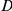 being the total number of observations (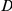 data), 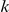 the number of possible values for 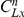 and 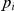 the number of observations of the specific value 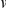 (that is, 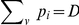). In other words, Laplace succession laws are very similar to histograms, except for the added terms at the numerator and denominator, that ensure that probabilities are never zero (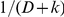 when 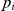 is 0) and that the initial form, before any observation, is a uniform probability distribution (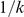 when 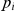 and 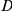 are 0).

The free parameters of the BAP model are thus the values of 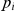 and 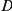, which are learned experimentally. In our implementation, the via-points variables are represented over discrete domains, with 41 integer values between 0 and 40 for position dimensions and 7 integer values between -3 and 3 for velocity dimensions. Consider 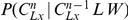, which is one of the terms of Eq. (4): it involves 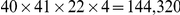 free parameters (for each writer, each letter, and each possible position of the previous via-point, a Laplace succession law of 41 parameters is defined over the current via-point position, but one of its parameter is not free because of the normalization rule).

Overall, the representation of letters involves a large database of free parameters: 8,960 for the first via-point, and, for each subsequent via-point, 296,032 free parameters. Notice that this number is much larger than the number of sample points in the learning database, which contains 3,520 trajectories. To solve this issue, the Laplace succession law probability distributions were smoothed using a binomial filter (of size 9 for position dimensions and size 7 for velocity dimensions) [47]. This allowed the generalization of experimental observations to neighboring, unobserved cases.

To help appreciate the gain in space requirement brought by the first-order Markov hypothesis, consider that the number of free parameters for each via-point would be 11,885,984 under a second-order Markov hypothesis, and 485,566,048 under a third-order Markov hypothesis. Concerning the separability of dimensions, for the 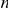-th via-point, the joint term 
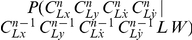
 would require 597,042,141,696 free parameters.

As a summary, the learning process amounts to counting, in the database, the number of observation of each case 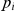: for each letter, writer and via-point position and velocity (index 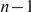), we obtained the number of observations 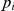 of each via-point position and velocity (index 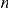). The result of this algorithm can be shown to be the maximum likelihood solution for the parameter of the BAP model, under the assumption of a uniform prior probability distribution over parameter values [48].

**Figure 9:**
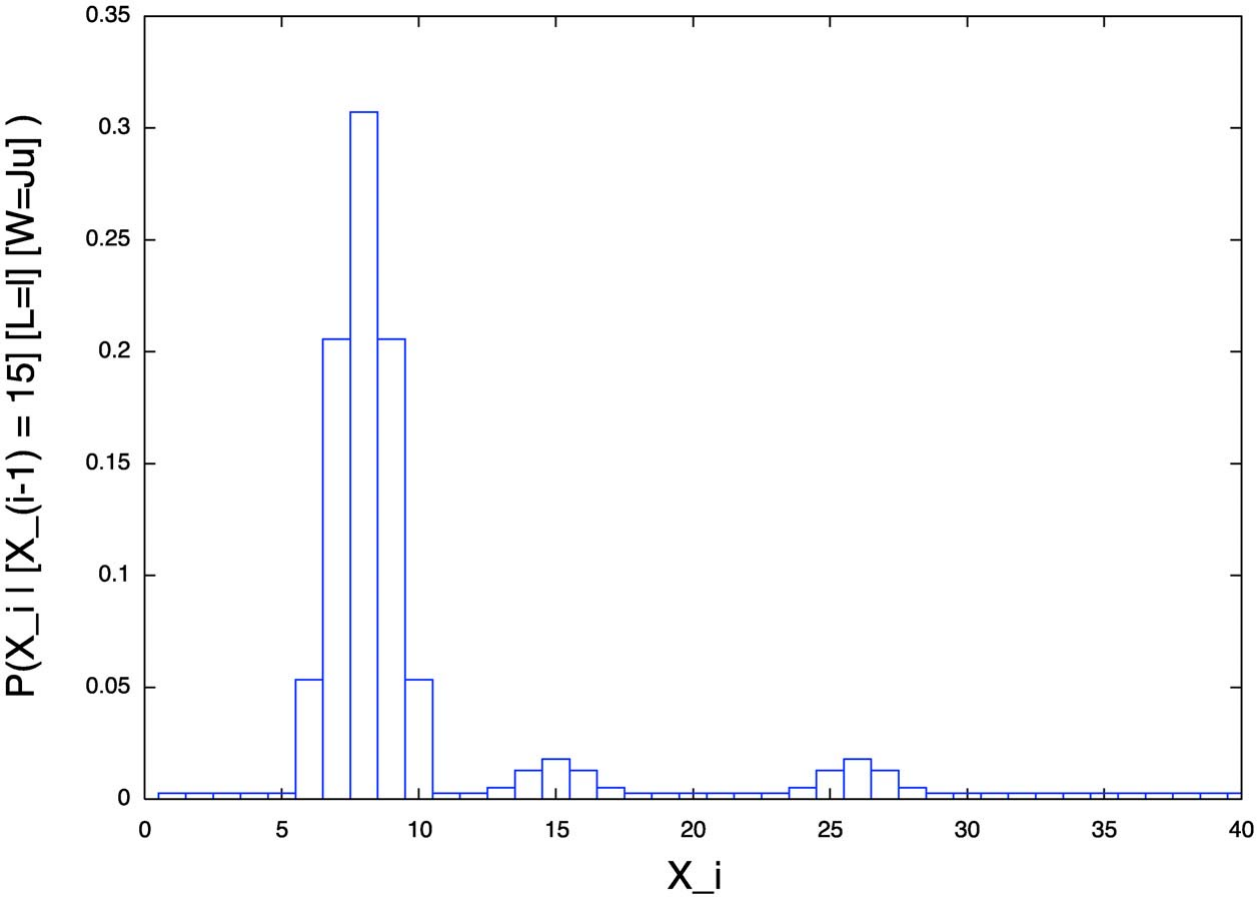
Example of probability distribution extracted from the learned model of letter representation. Probability distribution of the abscissa of the third via-point of the letter *l* and the writer 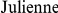, given that the abscissa of the second via-point is equal to 15: 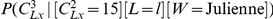.

Fig. 9 presents an example of a learned probability distribution for 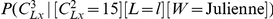: when the 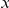 position of the second via-point is equal to 15, the 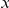 coordinate of the third via-point will probably be between 7 and 9. In other words, the third via-point is very likely to be on the left of the second via-point.

### Learned representations of letters: example

**Figure 10:**
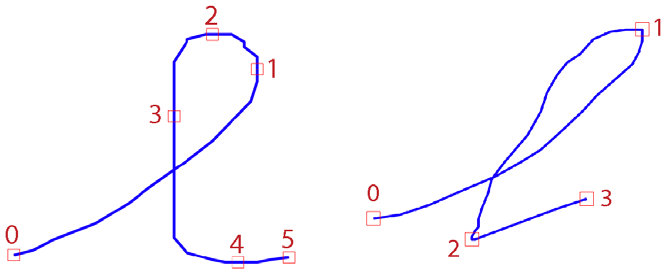
Two graphical forms of the same letter (*l*) written by the same writer 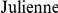. On the left, the letter is upward and described with 6 via-points. On the right, the slanted form yields only 4 via-points.

**Figure 11:**
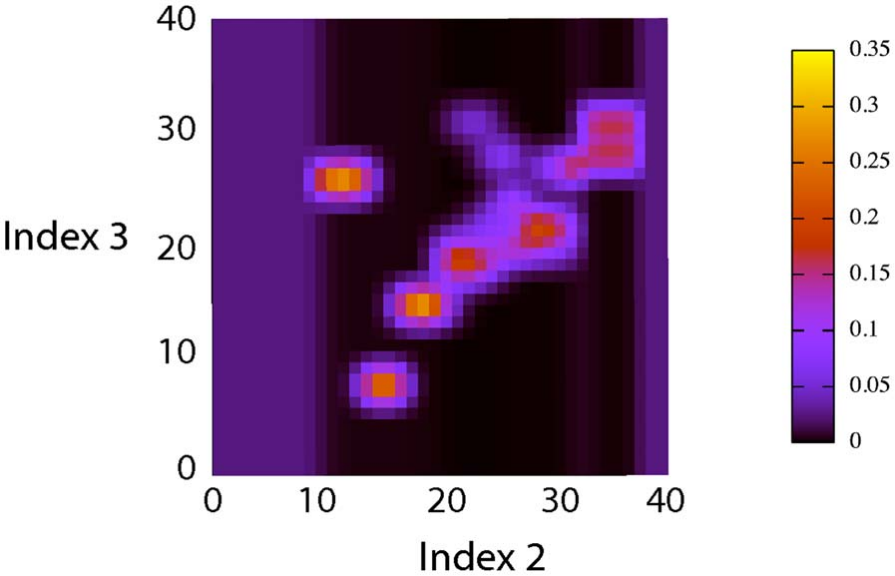
Example of several probability distributions extracted from the learned model of letter representation. Probability distributions of the abscissa of the third via-point of the letter (*l*) from the writer 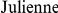, as a function of the abscissa of the second via-point: 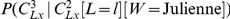. Each column is a probability distribution and sums to 1. For instance, Fig. 9 corresponds to the column for 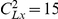.

Letters have many possible forms – called allographs – because of fluctuations in handwriting (see Fig. 10). The representation of letters must be robust to this within-writer variability. Indeed, the Laplace succession laws model this variability: they implicitly encode several allographs in one distribution. For instance, Fig. 11 presents the probability distribution of the third via-point of the letter *l*, given the position of the second via-point. The two allographs of Fig. 10 respectively correspond to the series of peaks below the diagonal (the third via-point is to the left of the second via-point, as in the upward *l*), and the main peak above the diagonal (the third via-point is to the right of the second via-point, as in the slanted *l*).

### Perception: reading letters

#### Question and inference

The cognitive task of letter recognition consists of identifying the presented letter. In other words, the question is: “given a trajectory produced by a known writer, what is the letter?” In probabilistic terms, this corresponds to computing:



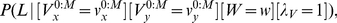
(21)


where 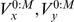 constitutes the input trajectory, 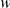 is the given writer, and 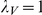 activates only the perception and letter representation parts of our model. Bayesian inference yields:



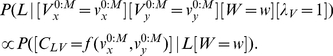
(22)


This probabilistic equation can be explained using an algorithmic equivalent. The computation proceeds as if the via-points extracted from the input trajectory were matched to the learned representations, for each letter. For each via-point and each possible letter, both positions and velocities are compared, using the memorized probability distributions: “if the letter was an *a* (*b*, *c*, etc.), what would be the probabilities of observing the positions and velocities of the first (second, third, etc.) observed via-point?”

**Figure 12:**
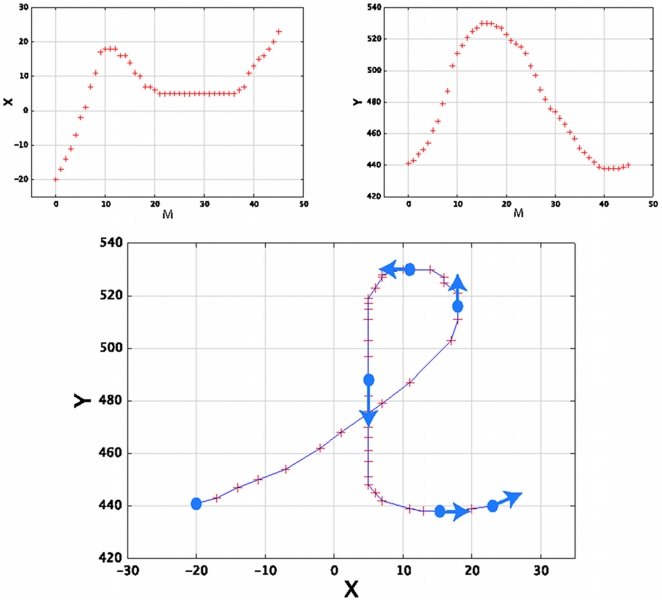
Example of input trajectory presented to the model for letter identification. It is composed of two series of 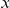 (top left) and 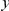 (top right) positions indexed by time, from which velocities are approximated using a finite difference method. Bottom: the trajectory presented in the 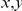 workspace, with extracted via-points superimposed (in blue). Vectors at the via-points represent velocity information.

For example, we computed the following question:



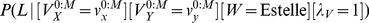
(23)


with 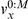, 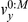 being the trajectory shown Fig. 12. On this particular example trajectory, the computed probability distribution is a Dirac distribution centered on 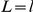: the model always correctly recognizes the input trajectory as being an *l*.

#### Results

We systematized the previous observations, in order to assess the quality of the letter recognition using a global recognition rate. To do so, we split our database of trajectories into a training set and a testing set, using 35 samples for training and 5 samples for testing. Training consisted of parameter identification, as previously described, and testing consisted of computing the probability distribution over letters 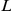 and using this distribution to draw randomly a value for 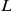. This selected value, the answer to the recognition task, was then used to assess whether the model had succeeded in recognizing the presented letter.

We repeated this procedure, varying the samples that were used for training and testing, applying classical K-fold cross-validation [49].

Overall, we obtained a satisfactory correct recognition rate of 93.36%. Misclassifications arose because of the geometric similarities of some letters. As an example, Table 1 shows an extract of the confusion matrix when the model is presented with examples of the letter *l*: we see that, overall, *l*s can be confused with *b*s or *h*s with low probabilities.

**Table 1:** Confusion matrix when the model is presented with examples of the letter *l*.

Letter	b	h	l
Probability	0.10	0.08	0.82

The letter *l* is recognized by the model with a probability of 0.82. It can be confused with a *b*, with probability 0.1, or an *h*, with probability 0.08. All probabilities for other letters are 0.

We extended our study of letter recognition by exploring several variants. Eq. (23) corresponds to the case where the writer is specified in the term to be computed. A slightly more difficult case is to hide this information from the model and to compute 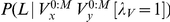 instead. The resulting Bayesian inference includes a summation over the missing variable, 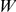. However, we still observed a high accuracy rate of 92.72%. An even more difficult case is to test letter recognition using the model with a new writer, by using testing trajectories provided by a writer who was not used in the training trajectories. In this case, the correct recognition rate drops to 49.68%.

#### Discussion

We now discuss the interpretation of the above recognition rates.

The first point here is to recall our objective. In an industrial application, it would make sense to find methods to improve the correct classification rates. However, in the context of modeling human perception–action loops, this is less of an issue. The above recognition rates are to be taken as performance predictions, which can then be compared with the predicted performance under other conditions. For instance, the basis for an experimental prediction is to compare the recognition performance with and without internal simulation of movements (see Section “Experimental predictions”).

Moreover, the recognition rates can only be compared assuming that the underlying learning databases are common. For a single test trajectory, the recognition process almost always outputs a probability distribution that is very close to a Dirac distribution. In other words, perception is almost always certain of its output, whether it leads to a correct or incorrect classification. Therefore, the correct classification rate mostly reflects the properties of the database contents: “how many test trajectories were similar enough to the learning trajectories to be correctly classified?”. The contrary would be a more objective, less contingent measure, such as “how close are cursive handwritten *g*s to *q*s?”, which would require a more systematic and complete database. Therefore, the obtained recognition rates mostly reflect the contents of the learning database, and not general properties of letters.

### Perception: recognizing writers

#### Question and inference

In the previous probabilistic question, a writer 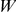 was specified, in order to compute a probability distribution over letters 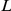. Reversing the role of these variables yields another perception task, which is writer recognition. Computing:



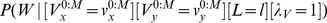
(24)


corresponds to building a probability distribution over writers, given an input trajectory and the identity of the presented letter.

This is solved by Bayesian inference in a manner similar to Eq. (22).

#### Results

Our model was tested once on 5 trajectory samples for each letter and each writer, taken from our database of 40 samples; the 35 remaining trajectories per letter and writer were used to identify, as previously, the parameters of the model. Because our database was small and specific, the global correct recognition rate of 79.5% mostly reflects idiosyncrasies of the writing styles of our 4 participants. However, Table 2 shows the full confusion matrix as a proof-of-concept example.

**Table 2:** Confusion matrix obtained for writer recognition.

	Estelle	Julienne	Jean-Louis	Christophe
Estelle	0.76	0.03	0.07	0.14
Julienne	0.02	0.80	0.07	0.11
Jean-Louis	0	0	1	0
Christophe	0.10	0.14	0.13	0.63

Probability distributions over the writers are read in rows: for instance, the model correctly identifies Estelle as the writer with probability 0.76, and Jean-Louis is always correctly identified (probability of 1).

As previously noted, when we made the letter recognition task more difficult by not specifying writer identity, we also tested writer recognition without specifying the letter identities. That is, we computed:



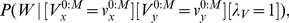
(25)


instead of Eq. (24). As previously mentioned, this yields a summation over the missing variable, which is 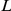 in this case. Experimental results show no qualitative change, with the recognition rate dropping from 79.5 to 78%.

Note that in this case, the writer is recognized independently of letter identification. In other words, the last variant we did not explore was joint writer and letter identification, which would have been solved by Bayesian inference by computing:



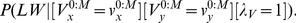
(26)


### Action: writing letters

We now turn to cognitive tasks that involve the action model of BAP. The main task developed here is simply the writing task.

#### Question and inference

Given a letter 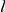 to write, and a writer 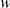 to imitate, what are the accelerations required to trace the letter? This writing task is translated, mathematically, by computing:



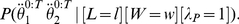
(27)


Bayesian inference yields:



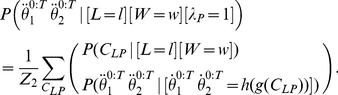
(28)


Instead of explicitly computing the costly summation of Eq. 28, we drastically approximate it, and, from this approximated probability distribution, sample acceleration values to apply to the simulated arm. This approximation can be seen as a two-step algorithm. First, the model of letter representation is used to draw randomly positions and velocities of via-points. Second, the trajectory generation model is used to determine the complete trajectory between the via-points. The effector model finally translates the Cartesian coordinates of points in the trajectory to joint coordinates and accelerations to apply.

Obviously, this only involves the motor branch and the representation of letter submodels: the perception branch is not used.

#### Results

**Figure 13:**
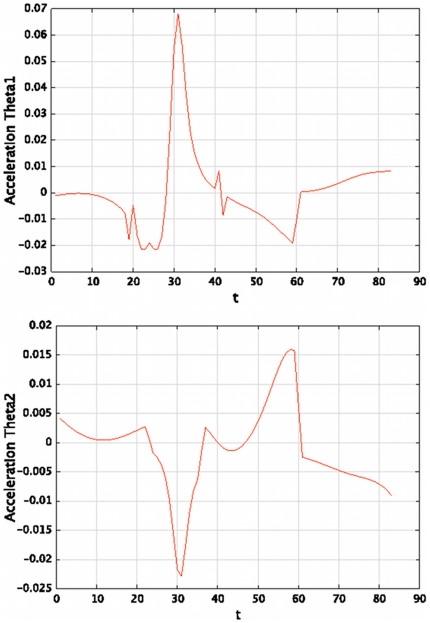
Example of joint accelerations computed by the model in order to generate a trajectory. Joint accelerations, 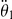 (top) and 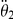 (bottom), as functions of time, obtained for the question 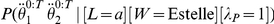.



Fig. 13 shows an example of acceleration profiles (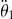 and 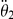) obtained in response to the question:

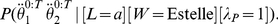
(29)


**Figure 14:**
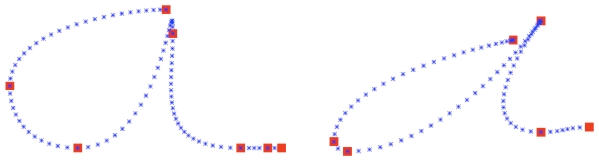
Examples of trajectories generated by the model, when asked to write an *a*. Left: the writing style specified is 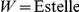. Right: 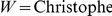.

“What are the accelerations to apply to the arm to write the letter *a* using the writing style of 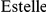
?” Applying these accelerations to the simulated arm yields the trajectory shown in Fig. 14 (left). This is readable and clearly identifiable as an *a*.

We now illustrate the fact that the BAP model reproduces between-writer variabilities. For instance, Fig. 14 shows trajectories for *a*s generated using the writing styles of Estelle and Christophe.

**Figure 15:**
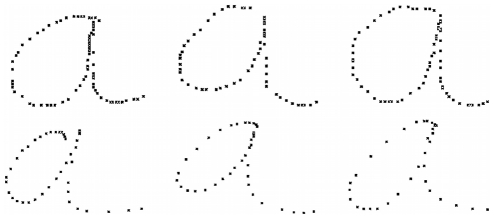
Illustration of between-writer variability present in the learning database. Top row: Three *a*s produced by Estelle. Bottom row: *a*s produced by Christophe. They are more slanted than Estelle's trajectories, which are more rounded.

We also compare the trajectories output by the model with typical trajectories provided by the participants in the database (see Fig. 15). We observe that writing styles are encoded and reproduced by the model. The *a*s produced by Estelle are typically rounded, whereas those produced by Christophe are more slanted and elongated.

**Figure 16:**
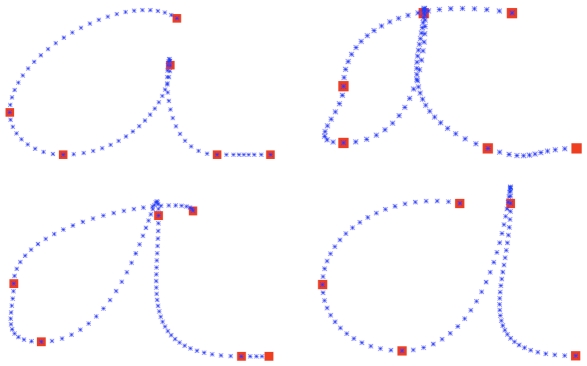
Within-writer inter-trial variability produced by the BAP model trajectory generation. Four trajectories obtained by computing 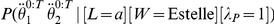.

Finally, if we ask the same question several times of the model, we observe within-writer intertrial variability; that is, the resulting trajectories are not identical (see Fig. 16). Indeed, as the positions and the velocities at via-points are drawn according to a probability distribution, the obtained trajectories vary. This result is, of course, in agreement with the everyday observation that every time we write, we are not producing exactly the same trajectory.

#### Discussion

We now wish to discuss two points about the above experiment: the origin of variability, and the observation of motor equivalence.

Firstly, our results show that the BAP model was able to reproduce both between-writer and within-writer variabilities. The question of variability in writing, and in motor control more generally, is crucial and presents a method for investigating the possible mechanisms involved. It is commonly agreed that variability does not result from a single step of the process. Perceptual processes (like target localization and proprioceptive feedback) and motor processes (like movement planning and movement execution) are assumed to contribute to observed variability [40], [50]–[52].

In the BAP model, movement planning is optimality-based and deterministic, the movement execution model is deterministic, and movements are simulated in an open-loop fashion. This would result in fixed produced trajectories [37], except that, in BAP, another source of variability exists, “upstream” of trajectory generation and execution. Indeed, at the representational level, positions and velocities at via-points are encoded by probability distributions; these yield the observed intertrial variability [37].

Finally, whereas most literature on the subject attributes motor variability to noise and “corruption” of the underlying processes, we would argue that variability is not always a nuisance. Indeed, in the BAP model, probability distributions at the representational level are multimodal and have high variances. This certainly yields variability in written trajectories, but it also provides generalization capabilities, between training examples of the limited database, which results in satisfying performance during letter recognition (93%).

We now turn to the motor equivalence effect, which motivated one of our founding hypotheses (see Section “Letter encoding in the Cartesian workspace”). This led us to assume that letters would be encoded in a Cartesian reference frame.

**Figure 17:**
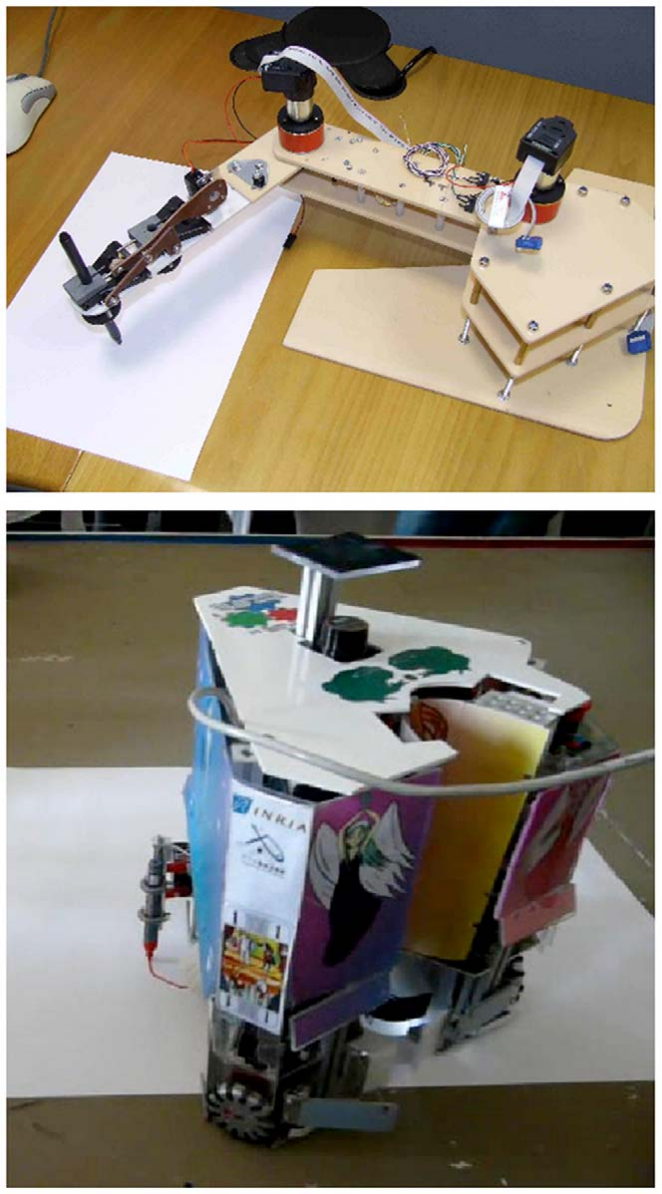
Two real robots used in our experiments. Top: two-joint robotic arm; Bottom: holonomic mobile platform. A pen is attached, and the robot moves and writes on large sheets of paper.

We experimentally verified that the BAP model satisfied motor equivalence. To do so, we used three different effectors to write letters and analyzed the resulting trajectories. The first was the simulated two-joint manipulator that we had used so far (see Fig. 8). The other two were real robotic devices: a real two-joint arm with the same characteristics as the simulated one, and a holonomic mobile robot (see Fig. 17).

These platforms required adapted effector models. Concerning the real robotic arm, it was built to be quite similar to the simulated one, except that it was controlled using velocity commands instead of acceleration commands. Therefore its effector model contains the first two terms of the simulated arm effector model (see Eq. (11)):



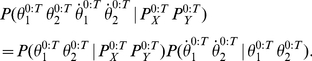
(30)


The holonomic mobile robot, on the other hand, is quite different from robotic arms. It is controlled by velocity commands to its three omnidirectional wheels, using a two-part effector model. First, from the 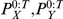 generated trajectory, a kinematic model computes velocity commands in the plane, 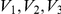, in each wheel's reference frame. Then, a control model translates these into lower-level rotation speeds 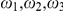. This is encoded in the following probabilistic effector model (the full definition of this model is available elsewhere [14]):



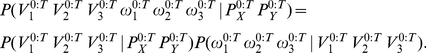
(31)


The first result of this experiment is that the three effectors correctly produce letters, given adequate effector models. In other words, no writing learning is required for the new effectors. Writing can be performed immediately on any new effector that we know how to control. This is in line with previous motor equivalence observations.

**Figure 18:**
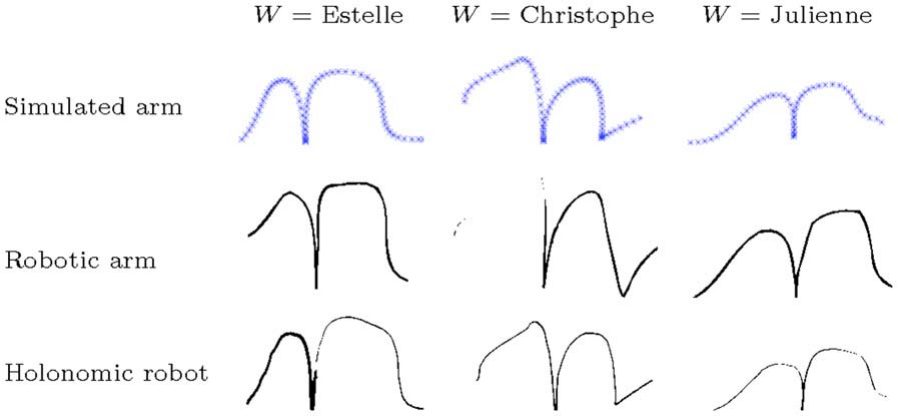
Examples of trajectories, illustrating the motor equivalence property. Trajectories produced by the BAP model simulating writers Estelle (left), Christophe (center), and Julienne (right), using a simulated arm (top row), a two-joint robotic arm (middle row) and a holonomic mobile robot (bottom row). Writing styles are preserved independently of the effector: notice, for example, the sharp peak at the end of the trajectory, which is specific to Christophe's writing style.

Furthermore, motor equivalence predicts that whichever effector is used, writing styles should be preserved and recognizable. We used the three effectors to write *n*s, imitating three different writers (see Fig. 18). We observe, as expected, recognizable characteristics in the trajectories, independent of the effector used to produce them.

### Perception and action: copying trajectories and letters

**Figure 19:**
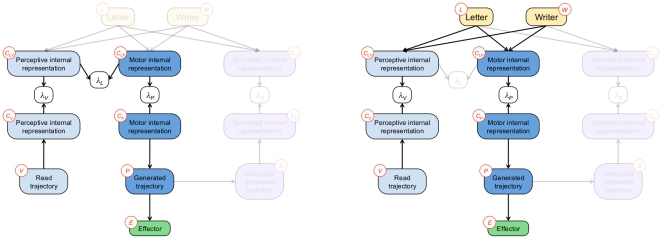
Copying trajectories and copying letters. Left: the highlighted parts of the model are activated during the copying of trajectories. Right: parts of the model involved in the copying of letters. Variables: 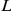 letter, 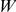 writer, 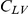 and 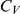 perceptive internal representations, 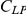 and 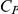 motor internal representations, 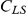 and 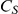 simulated internal representations, 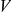 read trajectory, 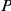 generated trajectory, 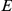 effector, 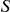 simulated generated trajectory, 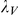, 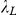, 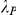 and 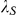 probabilistic switches. The probabilistic question corresponding to the copy of trajectories is shown Eq. (32), and the one for the copy of letters is shown Eq. (33).

We now turn to a cognitive task that involves the representation of letters, and the perception and action branches of the model. It consists in copying input trajectories. However, we distinguish the copies of trajectories, where the representations of letters are deactivated, from the copies of letters, where they are activated (see Fig. 19). The former can be seen as introductory to the last cognitive task of letter recognition with internal simulation of movement: copies involve the activation of most of the BAP model. We now detail each type of copy in turn.

### Perception and action: copying trajectories

#### Question and inference

In order to copy a trajectory, we provide an input trajectory and ask the model to compute the corresponding accelerations to apply to the simulated arm. This is translated mathematically and solved by Bayesian inference in the following manner:
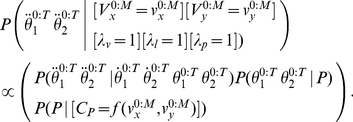
(32)


The term 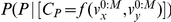 corresponds to the extraction of via-points from the perceived trajectory and to the generation of a full trajectory, based on these via-points. We observe that the model of letter representation is not involved in this question: the model does not analyze the trajectory in order to recognize the presented letter. As a consequence, with this mathematical translation of the task, any type of trajectory can be copied, not only those for known letters.

#### Results

**Figure 20:**
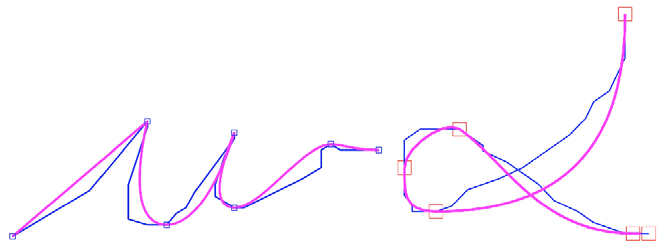
Examples of trajectory copying. The input trajectories are in blue; the copied, output trajectories are in pink.

We show, in Fig. 20, trajectories obtained with the copy-of-trajectories inference. The model can copy trajectories corresponding to known letters (e.g., *w*) and those corresponding to unknown symbols, outside of the learned repertoire (e.g., 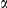).

We observe that the via-points extracted from the input trajectory are given directly as constraints to the trajectory generation. Input and output trajectories therefore coincide at the via-points, and the differences are situated between via-points.

### Perception and action: copying letters

#### Question and inference

The probabilistic question that corresponds to the copying of letters, and the resulting Bayesian inference, are:
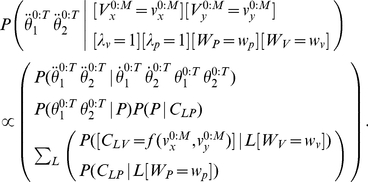
(33)


There are several differences between the questions for the copying of trajectories and the copying of letters. The first is that by not setting 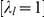 for the copying of letters (contrary to Eq. (32)), we no longer bypass the representation of letters. The second difference is that we modified the model slightly, by duplicating the writer variable 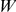 into 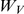 and 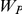, so that the input trajectory could be recognized as a letter according to the visual writer style, 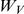, and be copied out according to another writer style, 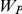.

The inference also appears more complicated for the copying of letters. However, it can be interpreted again as a schematically equivalent algorithm. Via-points are extracted from the input trajectory, and a probability distribution over recognized letters 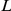 is computed. Given this probability distribution, a new sequence of via-points is drawn at random, which is a very rough approximation of the summation over 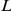 of Eq. (33). These are used to produce a new trajectory. Please note that at no point is the letter explicitly recognized: only the probability distribution over letters is computed.

#### Results

**Figure 21:**
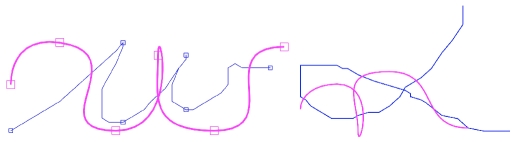
Examples of letter copying. The input trajectories are in blue; the output, copied trajectories are in pink. The model has recognized the trajectories as letters and has generated corresponding trajectories.

Fig. 21 presents results for the copying of letters.

We observe that the model has recognized and copied the letters (e.g., *w*). The trajectories produced by the model correspond to production of the recognized letters, in contrast to the copying of trajectories. Consequently, the graphical forms between input and output trajectories can be quite different, provided that the writing styles of the input and output writers are different. In other words, with this task, it is possible to copy a letter of a writer in the handwriting style of another writer (see, for instance, the *w* in Fig. 21).

Furthermore, when the presented trajectory does not correspond to a letter known by the model, the generated letter is the closest (in the sense of the probabilistic recognition of letters) in the known repertoire (e.g. an *n* instead of an 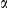).

#### Discussion

In the BAP model, two types of copying are formalized: when the letter representation is activated, letters are copied; when it is not, trajectories are copied. A question naturally follows: assuming that these two types of copying exist in humans, do their processes differ in the same way as in the model? Indeed, the model predicts that the difference is the use of the letter representation model. If that is the case, then preliterate children are only able to copy trajectories, and only older children and adults would be able to perform both tasks. Therefore, we should be able to observe a gradual appearance of letter copying, as the default process, as letter representations are acquired by children when they learn to read and write. This could possibly be challenging to observe experimentally in children, and could be instead investigated in adults, using letters of foreign alphabets or pseudo-characters to copy.

### Perception and action: reading letters using internal simulation of movements

The final cognitive task that we present revisits the reading of letters. While it was previously studied in a restricted version of our model, involving only the perception and representation of letters submodels, here we study the recognition of letters when the entire BAP model is activated. In other words, this task can also be seen as an extension of trajectory copying, where, instead of being executed, the planned trajectory is fed to the internal simulation of movement loop.

#### Question and inference

As previously discussed, we define the cognitive task of reading by the following question: “given an input trajectory, what is the corresponding letter?” We also provide, as input, the identity of the writer, although, as previously, this could be omitted in order to make the task more difficult. This task is translated and solved by Bayesian inference using the following:



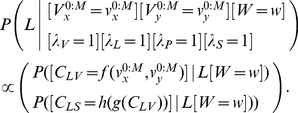
(34)


We observe that this equation is the product of two terms: 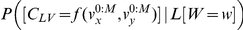 is exactly Eq. (22). In other words, this first term amounts to letter recognition in the reading task, where the motor and simulation parts of the model are not activated. This is the result of letter recognition where information only flows from the input trajectory to the representation of letters along the perception branch; via-points are extracted from the input trajectory and are compared with the memorized via-points for each letter.

The second term of Eq. (34) is 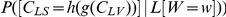. This also corresponds to letter recognition but using via-points that are the result of a longer circuit inside the model. First, via-points are extracted from the input trajectory, and then these are forwarded to the trajectory generation motor model, which generates a complete simulated trajectory. This is then forwarded to the simulated perception branch of the model, which extracts from it another set of via-points. These via-points are then compared in the letter representation model with the memorized letter representations.

An important point has to be recalled here. As discussed previously, we interpret the equations of Bayesian inference with pseudoalgorithms, using a few sentences. However, these would tend to suggest an ordered sequence of steps in the treatment of information, which does not follow at all from the original equations. For instance, in this case, the commutativity of the product obviously forbids the conclusion that the first term is computed before or after the second term. We believe this precaution is necessary, as it would be tempting, but wrong, to use the interpretations of the inferences to draw predictions about possible neural correlates. This is important in this case in particular because studying the properties of the internal simulations of movements during perception is a popular topic in neuroimagery [5], [13].

We also would like also to emphasize that it is not only temporal properties of the inference that require precaution. Just because schemas of our BAP model show spatially distinct subparts of models, this does not mean that we would expect spatially distinct corresponding areas in the central nervous system (CNS). More precisely, although we require mathematically distinct perception and simulated perception branches in the model, it could be the case that, in the CNS, there is only one set of areas that deal with both perception and simulated perception, with possibly temporally distinct or overlapping activations. If we correctly restrict ourselves to the algebraic notation, the model does not provide any prediction about spatial or temporal properties of possible neural correlates.

#### Results

**Figure 22:**
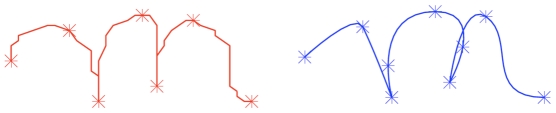
Two trajectories are used in the reading task, when internal simulation of movements is activated. Left: input trajectory presented to the model, and corresponding via-point positions. Right: Internally simulated trajectory produced by the model, and resulting via-points extracted by simulated perception.

Firstly, we present an illustrative result. Fig. 22 shows the two main trajectories involved in the reading task using internal simulation: the input trajectory and via-points extracted by the perception branch, and the internally simulated trajectory and via-points that result from simulated perception.

We observe that the sets of via-points shown for perception and simulated perception are not identical (see, for instance, the via-points along the middle arc, which are added by internal simulation). Letter recognition uses both sets of via-points; in this example, the letter is recognized as an *m* with probability 1.

Secondly, we present systematic evaluations of our results. We tested the model under the same conditions as in the reading task using only the perception submodel (see Section “Perception: reading letters”) and obtained an overall recognition rate of 90.22%. An analysis of the confusion matrices of both experiments (not shown) indicates that specific errors differ; some letters that were misclassified in the reading task without simulated perception were correctly recognized using simulated perception, and vice versa. However, the overall misclassification rates are of the same magnitude under both conditions (90 vs. 93%).

Finally, because of the similar observed performance between reading with motor simulation and reading without motor simulation under classical conditions, we have designed another experiment with a more difficult scenario. Instead of presenting complete trajectories as inputs, we designed truncated versions of trajectories where we erased a set of consecutive points.

**Figure 23:**
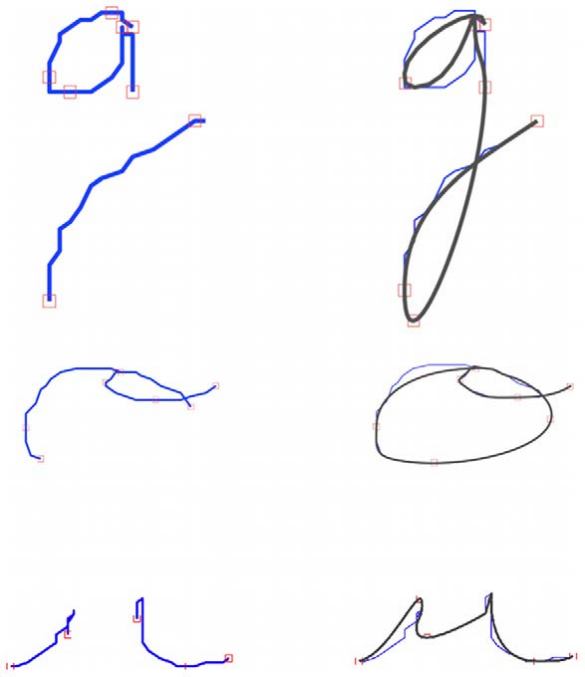
Example cases where the internal simulation of movements helps identify the correct letter. Left: incomplete trajectories presented to the model (in blue) and corresponding via-points extracted by perception. Right: the same trajectory with the trajectory generated by internal motor simulation (in black) and resulting via-points extracted by simulated perception (in red).

We have found several cases where reading without motor simulation would fail but reading with motor simulation would succeed. We illustrate here a few of such cases, shown in Fig. 23.

Consider, for instance, the *g*s shown at the top of Fig. 23. Table 3 shows extracts of the probability distributions over letters obtained by reading with and without internal simulation. We observe that the incomplete *g* is misclassified as a *q* without simulation, and correctly recognized as a *g* with internal simulation of movements. In this case, we can conclude that internal simulation of movements helps improve stimulus recognition.

**Table 3:** Extracts of the probability distributions over letters, with and without motor simulation.

	f	g	h	k	l	m	n	o	p	q	r
With motor simulation	0	0	0	0	0	0	0	0	0	1	0
Without motor simulation	0	0.9	0	0	0	0	0	0	0	0.1	0

Extracts of the probability distributions over letters, computed as solutions to the reading task with (top row) and without (bottom row) motor simulation, when presented with the truncated *g* shown Fig. 23.

Recall that we only consider isolated letters, so that contextual and semantic cues are not available and thus not modeled in BAP. Had they been available, an open issue would have been to model the combination of these top-down cues with the bottom-up cues that we showed were provided by motor simulation.

#### Discussion

We wish to conclude this section with a speculative consideration. We have shown that internal simulation could have a major effect during perception. For instance, it is able to overcome the difficult case of incomplete input trajectories. Another way to make the perception task harder would be to remove sequence information: we would then consider offline letter recognition, instead of online letter recognition as we have done so far.

We believe that in this case, internal simulation of movements might also be very beneficial. It is known that writing expertise, in particular the directions of commonly performed movements, biases perception [3]. We would therefore imagine that when seeing a written *l*, motor knowledge of stereotypical movements would help in selecting the starting point and sequence of the trajectory. The offline problem would first be reduced to the online problem that we have addressed in this paper. This seems easy if assuming motor knowledge but appears a very difficult step for a purely perception-based system (i.e., one with trajectory and sequence recovery from a pixel-based image).

## Discussion

### Summary

In this paper, we defined a Bayesian model of the perception–action loop involved in handwriting and letter recognition, and used it to study the influence of motor simulation on perception.

Four hypotheses form the basis of the architecture of the BAP model. First, there are two distinct internal representations of letters: one for the perception model and one for the action model. Second, these representations are of the same nature (Cartesian space) and are based on the same encoding. Third, this encoding consists in summarizing letter trajectories by sequences of via-points, which lie at points where the tangent is either vertical or horizontal, and at cusps. Finally, a feedback loop from the generated trajectories back to the internal representation of letters implements an internal simulation of movements.

We used probabilistic modeling to define the BAP model mathematically. With the joint probability distribution fully defined, the model was used to solve cognitive tasks automatically, using Bayesian inference. We detailed six such tasks in the paper: reading with and without motor simulation, writer recognition, copying of letters and trajectories, and writing letters with different effectors. In particular, we showed that internal simulation of movements improves performance for reading tasks in the difficult case of truncated letters.

### General discussion

#### Related work

In Cognitive Science, Bayesian modeling of perception and action is blooming, but the modern trend yields models that differ somewhat in flavor from the BAP model we presented here. A wide variety of domains has been explored with probabilistic modeling approaches; to name just a few examples, visuo-haptic fusion [53], visual perception [54] and motor control [40]. These mainly come from experimental psychophysics and psychology.

In contrast, our approach draws inspiration from research in robotics and artificial intelligence [13], [48], [55]. Several models of cognitive systems, like the BAP model we presented here, have been developed in that context. They range from eye movement selection [56] to self-motion perception [57] and speech acquisition [58]. Their main feature is that they are structured models, as a pendant to classical computer science structured programs. This contrasts with the previously cited approaches that mostly focus on “flat” models, with a single likelihood function and a – usually informed – probabilistic prior distribution.

The recent “causal inference” model [59]–[61] is noteworthy, in this regard, as it appears to stand halfway between the two approaches: an internal, latent variable represents the number of presented sources and weighs the relative contributions of two sub-models. Each model perception under the assumption that there is either one or two sources.

To get a more in-depth presentation of how all these models, and other, relate, the interested reader can refer to a recent article in which we proposed a treatment of several usual cognitive issues [62]. In this paper, we show how probabilistic models of these cognitive issues can be formally rewritten and cast in the notation of Bayesian Programming, which then serves as a unifying framework. (Formally, the class of models written in the Bayesian Programming notation is the same as the class of models that can be written using probabilistic factor graphs. This is a proper superset of the models that can be written using Bayesian Networks.) This is presented using an incremental traversal of several Bayesian models, highlighting the corresponding graduation in structure complexity.

Concerning the BAP model, it is easily seen that it also heavily features structured modeling. Indeed, the two central hypotheses of BAP are related to its structure. Firstly, we assumed a representation of letter that acts as pivot between perception and action processes. Secondly, we assumed an internal loop implementing a simulation of movement preparation and perception. Conditional independence hypotheses also have been used (see the dimension separability and Markov hypothesis of Eq. (4)), simplifying the BAP model structure and making it computationally tractable.

The main advantage, we believe, of the proposed approach lies in its expressive power. In this view, Bayes' rule is not restricted to deal with the combination of prior probability distributions about hypotheses and likelihood functions about observations. We apply Bayes' rule instead to combine various representations, and conditional independence hypotheses to structure the relations between these representations. In that regard, our approach is clearly to be considered part of the “algorithmic” level of Marr's hierarchy of levels of analysis [63]. Alternatively, to use the vocabulary of a recent discussion, it is part of the Bayesian Enlightenment school of probabilistic modeling [64].

Of course, this expressive power comes at a price: experimental validation of complex models with large number of parameters is not easy. In previous research, we have validated models by showing they closely predicted a large variety of experimental results in the literature [65], independently of the fine tuning of internal parameters. Another approach, inspired by psychophysics and widely applied in related research, is to first calibrate the model parameters on some experimental data of control conditions, and then validate the model by its ability to predict observations for test conditions. A third approach consists in defining variants of a model and comparing their adequacy to some experimental data using Bayesian model comparison [56], [66]. Because of the complexity of the BAP model, and its large number of internal parameters, such methods are inadequate for the BAP model. Therefore, we explored another approach: all parameters being set, we compared predictions for a few variants of the model. This allows the study of general properties of structures of the variants under scrutiny. For instance, we studied letter recognition both with and without activation of the internal simulation of movements.

#### Experimental predictions

Indeed, because the translation of assumptions into the model is so transparent in the probabilistic framework, the BAP model can be seen as a basis; variants of BAP can easily be created, and their performance can be analyzed in various numerical simulations.

For instance, we have assumed that position and velocity information was encoded at via-points, as suggested by previous experiments [67]. It is then straightforward to remove, from the current BAP formulation, velocity information from the model, and to simulate both versions concurrently for a variety of tasks, looking for situations where predictions are different. Moreover, the Bayesian formalism easily accommodates the systematic search of such distinguishable predictions [68].

The BAP model is also a fertile ground for producing testable hypotheses in a more classical manner. Some properties of the BAP model, as described in this paper, can already be the basis of experimentation. We highlight one example here, which is probably the most central to the current paper.

Consider the internal simulation of movement loop, and its use in perception tasks. We have shown that, under the condition of complete letters, the recognition rate was similar between the cases where perception was based purely on sensory processing and where it was complemented by simulated perception. However, under the condition of truncated letters, we have shown examples where pure perception would fail, whereas perception with the internal simulation of movements would recover information so as to identify the stimulus correctly.

In other words, there is a predicted interaction between the difficulty of the recognition task and the use of internal simulation. There are a number of ways to limit the use of motor simulation experimentally; for example, the widespread Transcranial Magnetic Stimulation (TMS) technique. However, there are also more low-tech solutions, such as interfering with the motor processes by introducing a concurrent motor task. This has recently been shown to be effective in a variety of situations, such as distance perception [69] or letter recognition [4].

Based on this predicted interaction, we designed and are currently running an experiment.

#### Learning representations of letters

As we have previously argued, we believe that the BAP model, thanks to its mathematical formulation, is a basis for exploring the properties of variants, in order to study the relevance of underlying hypotheses.

Finally, we also argue that it is the basis for developing other aspects of human letter perception and production, which we have so far left out of our scope. Consider, for instance, the learning of reading and writing.

In BAP, we have treated the learning of the model parameters in a mathematically straightforward yet highly implausible manner. For instance, the trajectories of the learning database are fed in a single batch to the internal representation submodel, which computes its parameter accordingly. We have also assumed that motor control, and more precisely the general purpose effector model, was available directly from the start and was highly accurate (i.e., the effector and trajectory formation submodels are deterministic, with no control noise and uncertainties added).

In other words, the BAP model, as presented in this paper, can be seen as a highly skilled painter, adult and illiterate, who would learn how to read by observing many letter samples and then immediately be able to write as well as an expert. This is obviously a model of a very specific and improbable case.

It is more usual for children to learn reading, writing, and motor control simultaneously.

This implies that perceptual samples come from external sources and also from early trials of the production of letters. Whether this would help or hinder the formation of suitable internal representations of letters is an open question, which would be relevant to the study of the pedagogy of writing.

This question could also be tackled by exploiting a leverage that we have not used in the current paper: even though the perceptive and motor internal representation models are duplicated in BAP, their content is so far always identical. The question of whether they could be collapsed into a single representation, or whether duplicate and coconstructed representations are needed, is still open.

## Supporting Information

Appendix S1 (“Activation or deactivation of submodels: the Bayesian switch”) is provided as a supplementary material. It describes the formal definition of Bayesian switches in the general case, and the mathematical proof that some inferences yield activation or deactivation of submodels it connects, thus making it behave like a switch between submodels.
(PDF)

## References

[1] Orliaguet JP, Kandel S, Boë LJ (1997). Visual perception of motor anticipation in cursive handwriting: Inuence of spatial and movement information on the prediction of forthcoming letters.. Perception.

[2] Knoblich G, Seigerschmidt E, Flach R, Prinz W (2002). Authorship effects in the prediction of handwriting strokes: Evidence for action simulation during action perception.. The Quarterly Journal of Experimental Psychology.

[3] Li JL, Yeh SL (2003). Do “Chinese and American see opposite apparent motions in a Chinese character”? Tse and Cavanagh (2000) replicated and revised.. Visual Cognition.

[4] James KH, Gauthier I (2009). When writing impairs reading: letter perception's susceptibility to motor interference.. Journal of Experimental Psychology: General.

[5] Longcamp M, Anton JL, Roth M, Velay JL (2003). Visual presentation of single letters activates a premotor area involved in writing.. NeuroImage.

[6] Longcamp M, Tanskanen T, Hari R (2006). The imprint of action: Motor cortex involvement in visual perception of handwritten letters.. NeuroImage.

[7] Hollerbach JM (1981). An oscillation theory of handwriting.. Biological Cybernetics.

[8] Edelman S, Flash T (1987). A model of handwriting.. Biological Cybernetics.

[9] Meulenbroek RGJ, Rosenbaum DA, Thomassen AJW, Loukopoulos LD, Vaughan J (1996). Adaptation of a reaching model to handwriting: How different effectors can produce the same written output, and other results.. Psychological Research.

[10] Crettez JP, Lorette G (1998). Reconnaissance de l'écriture manuscrite. traité Informatique..

[11] Vuori V (2002). Adaptive Methods for On-Line Recognition of Isolated Handwritten Characters..

[12] Lebeltel O, Bessière P, Diard J, Mazer E (2004). Bayesian robot programming.. Autonomous Robots.

[13] Bessière P, Laugier C, Siegwart R (2008). Probabilistic Reasoning and Decision Making in Sensory-Motor Systems, volume 46 of *Springer Tracts in Advanced Robotics.*.

[14] Gilet E (2009). Modélisation Bayésienne d'une boucle perception-action: application à la lecture et à l'écriture..

[15] Prinz W (1997). Perception and action planning.. European Journal of Cognitive Psychology.

[16] Liberman AM (1957). Some results of research on speech perception.. Journal of the Acoustical Society of America.

[17] Liberman AM, Mattingly IG (1985). The motor theory of speech perception revised.. Cognition.

[18] Schwartz JL, Abry C, Boë LJ, Cathiard M (2002). Phonology in a theory of perception-for-actioncontrol..

[19] Bernstein N (1967). The Co-ordination and Regulation of Movements..

[20] Serratrice G, Habib M (1993). L'écriture et le cerveau..

[21] Wright CE, Charles WrightE, Jeannerod M (1990). Generalized motor programs: Reexamining claims of effector independence in writing.. Attention and Performance XIII: Motor Representation and Control.

[22] Anquetil E, Lorette G (1995). Reconnaissance en ligne de lettres manuscrites cursives par chaînes de markov cachées.. Traitement du signal.

[23] Artieres T, Marukatat S, Gallinari P (2007). Online handwritten shape recognition using segmental hidden markov models.. IEEE Trans Pattern Anal Mach Intell.

[24] Yacoubi AE (1996). Modélisation markovienne de l'écriture manuscrite, application à la reconnaissance des adresses portales.. Ph.D. thesis, Université de Rennes 1.

[25] Schomaker L, Teulings HL, Helsper E, Abbink G (1993). Adaptive recognition of online, cursive handwriting.. Proceedings of the Sixth International Conference on Handwriting and Drawing.

[26] Wada Y, Kawato M (1995). A theory for cursive handwriting based on the minimization principle.. Biological Cybernetics.

[27] Wada Y, Koike Y, Vatikiotis-Bateson E, Kawato M (1995). A computational theory for movement pattern recognition based on optimal movement pattern generation.. Biological Cybernetics.

[28] Cooper LA, Shepard RN (1973). The time required to prepare for a rotated stimulus.. Memory & Cognition.

[29] Jeannerod M (2001). Neural simulation of action: A unifying mechanism for motor cognition.. NeuroImage.

[30] Berthoz A (2000). The Brain's Sense of Movement..

[31] Calvo-Merino B, Glaser D, Grèzes J, Passingham R, Haggard P (2004). Action observation and acquired motor skills: An fMRI study with expert dancers..

[32] Bengio Y, Frasconi P, Tesauro G, Touretzky D, Leen T (1995). An input/output HMM architecture.. Advances in Neural Information Processing Systems 7.

[33] Murphy K (2002). Dynamic Bayesian Networks: Representation, Inference and Learning..

[34] Wolpert D, Ghahramani Z, Jordan M (1995). An internal model for sensorimotor integration.. Science.

[35] Ghahramani Z, Wolpert D (1997). Modular decomposition in visuomotor learning.. Nature.

[36] Paillard J, Sirat C, Irigoin J, Poulle E (1990). Les bases nerveuses du contrôle visuo-manuel de l'écriture..

[37] Todorov E (2004). Optimality principles in sensorimotor control.. Nature Neuroscience.

[38] Flash T, Hogan N (1985). The coordination of arm movements: An experimentally confirmed mathematical model.. Journal of Neuroscience.

[39] Uno Y, Kawato M, Suzuki R (1989). Formation and control of optimal trajectory in human multijoint arm movement - minimum torque-change model.. Biological Cybernetics.

[40] Harris CM, Wolpert DM (1998). Signal-dependent noise determines motor planning.. Nature.

[41] Siciliano B, Sciavicco L, Villani L, Oriolo G (2009). Robotics – Modelling, Planning and Control. Advanced Textbooks in Control and Signal Processing Series..

[42] Desmurget M, Jordan MI, Prablanc C, Jeannerod M (1997). Constrained and unconstrained movements involve different control strategies.. Journal of Neurophysiology.

[43] Bessière P, Ahuactzin JM, Aycard O, Bellot D, Colas F (2003). Survey: Probabilistic methodology and techniques for artefact conception and development..

[44] Cooper G (1990). The computational complexity of probabilistic inference using bayesian belief networks.. Artificial Intelligence.

[45] Bessière P (2004). Method for determining a value given to different parameters of a system.. WO Patent WO/2004/013,714.

[46] Mekhnacha K, Ahuactzin JM, Bessière P, Mazer E, Smail L (2007). Exact and approximate inference in ProBT.. Revue d'Intelligence Artificielle.

[47] Aubury M, Luk W (1996). Binomial filters.. Journal of VLSI Signal Processing.

[48] Jaynes ET (2003). Probability Theory: The Logic of Science..

[49] Russell S, Norvig P (1995). Artificial Intelligence: A Modern Approach..

[50] Guigon E, Baraduc P, Desmurget M (2008). Computational motor control: feedback and accuracy.. European Journal of Neuroscience.

[51] van Beers RJ, Baraduc P, Wolpert DM (2002). Role of uncertainty in sensorimotor control.. Phil Trans R Soc Lond B.

[52] van Beers RJ, Haggard P, Wolpert DM (2004). The role of execution noise in movement variability.. Journal of Neurophysiology.

[53] Ernst M, Banks M (2002). Humans integrate visual and haptic information in a statistically optimal fashion.. Nature.

[54] Kersten D, Mamassian P, Yuille A (2004). Object perception as bayesian inference.. annu Rev Psychol.

[55] Thrun S, Burgard W, Fox D (2005). Probabilistic robotics..

[56] Colas F, Flacher F, Tanner T, Bessière P, Girard B (2009). Bayesian models of eye movement selection with retinotopic maps.. Biological Cybernetics.

[57] Laurens J, Droulez J (2007). Bayesian processing of vestibular information.. Biological Cybernetics.

[58] Serkhane J, Schwartz JL, Bessière P (2005). Building a talking baby robot: A contribution to the study of speech acquisition and evolution.. Interaction Studies.

[59] Körding KP, Beierholm U, Ma WJ, Quartz S, Tenenbaum JB (2007). Causal inference in multisensory perception.. PLoS one.

[60] Sato Y, Toyoizumi T, Aihara K (2007). Bayesian inference explains perception of unity and ventriloquism aftereffect: Identification of common sources of audiovisual stimuli.. Neural Computation.

[61] Shams L, Beierholm UR (2010). Causal inference in perception.. Trends in Cognitive Science.

[62] Colas F, Diard J, Bessière P (2010). Common bayesian models for common cognitive issues.. Acta Biotheoretica.

[63] Marr D (1982). Vision. A Computational Investigation into the Human Representation and Processing of Visual Information..

[64] Jones M, Love B Bayesian fundamentalism or enlightenment? on the explanatory status and theoretical contributions of bayesian models of cognition..

[65] Colas F, Droulez J, Wexler M, Bessière P (2007). A unified probabilistic model of the perception of three-dimensional structure from optic ow.. Biological Cybernetics.

[66] Daunizeau J, den Ouden HEM, Pessiglione M, Kiebel SJ, Friston KJ (2010). Observing the observer (II): Deciding when to decide.. PLoS one.

[67] Kandel S, Orliaguet JP, Boë LJ (2000). Detecting anticipatory events in handwriting movements.. Perception.

[68] Diard J (2009). Bayesian model comparison and distinguishability.. Proceedings of the International Conference on Cognitive Modeling (ICCM 09).

[69] Witt JK, Profftt DR (2008). Action-specific inuences on distance perception: A role for motor simulation.. Journal of Experimental Psychology: Human Perception and Performance.

