# Supplementary material for: Bayesian Action–Perception Computational Model: Interaction of Production and Recognition of Cursive Letters
Source: PLoS One. 2011 Jun 1;6(6):e20387. doi: 10.1371/journal.pone.0020387 (PMC3106017; doi:10.1371/journal.pone.0020387)
Supplement: Appendix S1 — (“Activation or deactivation of submodels: the Bayesian switch”) is provided as a supplementary material. It describes the formal definition of Bayesian switches in the general case, and the mathematical proof that some inferences yield activation or deactivation of submodels it connects, thus making it behave like a switch between submodels. (PDF) [file pone.0020387.s001.pdf]

Appendix S1: Activation or deactivation of submodels: the Bayesian switch

Let A and B be probabilistic variables that share the same domain $\mathcal{D}$. Let λ be a binary variable, taking 0 or 1 values. We show here how λ can be used as a "probabilistic switch" between variables A and B.

We define the joint probability distribution as follows:

$$P(A\ B\ \lambda) \quad = \quad P(A)P(B)P(\lambda \mid A\ B) \ .$$

The terms $P(A)$ and $P(B)$ can be arbitrarily defined, and $P(\lambda \mid A\ B)$ is defined by:

$$P([\lambda = 1] \mid [A = a]\ [B = b]) \quad = \quad \begin{cases} 1 \text{ if } a = b \\ 0 \text{ otherwise} \end{cases}$$

Firstly, consider $P([A = a])$. It can be trivially shown that it is simply $P(A)$ as defined in the model. By assumption, it is thus independent of $P(B)$.

Secondly, consider $P([A = a] \mid [\lambda = 1])$:

$$\begin{aligned} & P([A = a] \mid [\lambda = 1]) \\ & \quad = \quad \frac{P([A = a]\ [\lambda = 1])}{P([\lambda = 1]} \\ & \quad \propto \quad \sum_{B} P([A = a])P(B)P([\lambda = 1] \mid [A = a]\ B) \ . \end{aligned}$$

In the above summation, there is a single value of B for which $P([\lambda = 1] \mid [A = a]\ B)$ is not zero: when $B = a$. Therefore the summation collapses:

$$\begin{aligned} & P([A = a] \mid [\lambda = 1]) \\ & \quad \propto \quad P([A = a])P([B = a])P([\lambda = 1] \mid [A = a]\ [B = a]) \\ & \quad \propto \quad P([A = a])P([B = a]) \ . \end{aligned}$$

Therefore, $P([A = a] \mid [\lambda = 1])$ is not independent of $P(B)$, contrary to $P([A = a])$. When λ is specified in the right-hand side of the computed term, B is forced to take the value taken by A.

In this manner, λ can be used as a switch. When we want the submodels about A and B to be disconnected, λ is not specifed; from the point of view of B, A is deactivated. On the other hand, if we want submodel A to influence submodel B, we specify $\lambda = 1$ in the questions we compute, and both submodels are activated.
